# Pullulan Fatty Ester Nanocoatings with Tunable Surface Wettability and Bio-Inert Surface Properties

**DOI:** 10.3390/ijms27177768

**Published:** 2026-08-30

**Authors:** Femke De Ceulaer, Hao-Chun Chiu, Olivier Deschaume, Carmen Bartic, Pedro Fardim

**Affiliations:** 1 Chemical and Biochemical Reactor Engineering and Safety (CREaS), Department of Chemical Engineering, Katholieke Universiteit Leuven (KU Leuven), Celestijnenlaan 200F, 3001 Leuven, Belgium; 2 Laboratory for Soft Matter Physics and Biophysics, Department of Physics and Astronomy, Katholieke Universiteit Leuven (KU Leuven), Celestijnenlaan 200D, P.O. Box 2416, 3001 Leuven, Belgium

**Keywords:** pullulan, fatty acid, esterification, hydrophobic, surface properties

## Abstract

Polysaccharide derivatives with tunable hydrophobicity offer a versatile platform for tailoring material properties through controlled chemical modification. In this study, pullulan was functionalized with saturated fatty acids (C14-C18) via N,N-carbonyldiimidazole-mediated esterification, yielding degrees of substitution between 0.24 and 0.77. The effect of alkyl chain length and substitution levels on the thermal, structural, and surface properties of the resulting pullulan esters was systematically investigated. Increasing chain length enhanced thermal stability and promoted structural ordering, with stearate-modified pullulan exhibiting melting transitions at 40–42 °C and higher degradation temperatures (≥312 °C). Spin-coated nanoscale coatings (110–150 nm thick) exhibited extremely smooth surfaces (S_a_ < 1 nm), although differences in solubility influenced solvent evaporation and induced nanostructural variations. Surface wettability increased systematically with alkyl chain length and degree of substitution, resulting in water contact angles between 85° and 105°. All coatings were non-cytotoxic and inhibited bacterial growth at the interface without leaching, suggesting a contact-dependent antibacterial effect. These results establish clear structure–property relationships in pullulan esters, demonstrating that surface wettability, structural ordering, and surface functionality can be systematically adjusted via alkyl chain length and degree of substitution.

## 1. Introduction

Polysaccharides have increasingly attracted attention over recent decades as building blocks for advanced functional materials due to their inherent biocompatibility, chemical tunability, and film-forming capability [1]. Among these materials, the linear and flexible natural polysaccharide pullulan, derived from the fungus *Aureobasidium pullulans*, is particularly promising because of its excellent film-forming properties and pH-independent water solubility. Pullulan is biocompatible, biodegradable, non-toxic, non-immunogenic, and non-carcinogenic, making it highly suitable for biomedical and materials applications [2,3,4]. Structurally, it consists of repeating maltotriose units linked via α-(1,6) glycosidic bonds, where each maltotriose contains three glucose units connected by α-(1,4) glycosidic linkages. The presence of nine hydroxyl groups per repeating unit provides extensive opportunities for chemical modification [2,3,5,6].

The introduction of hydrophobic moieties into polysaccharides represents an effective strategy to tailor their physicochemical properties by modifying intermolecular interactions and molecular organization [7]. In particular, esterification with fatty acids of varying alkyl chain length enables systematic tuning of solubility, thermal transitions, and material behavior through changes in chain packing and side-chain interactions [8,9,10,11]. While these modification strategies have been extensively studied for polysaccharides such as cellulose, starch, and dextran, their behavior is strongly influenced by the intrinsic structure of the polymer backbone [8,11,12,13,14]. Cellulose consists of a rigid β-(1,4)-linked structure that forms highly ordered packing arrangements, which affect chain mobility and accessibility of hydroxyl groups [15]. In contrast, starch exhibits a semi-crystalline architecture with both amorphous and crystalline domains, while dextran consists of a predominantly α-(1,6)-linked backbone with a variable degree of branching, depending on its origin and synthesis conditions [16,17,18]. This structural variability leads to irregular chain packing and limits the formation of well-defined crystalline domains. Unlike these polysaccharides, pullulan is a linear α-(1,4)- and α-(1,6)-linked polymer that is fully amorphous and lacks long-range structural order [19]. Consequently, its intermolecular interactions and packing behavior upon chemical modification are expected to differ substantially.

Esterification with fatty acids has been widely investigated for cellulose, starch, and dextran, where the effects of alkyl chain length and degree of substitution (DS) on thermal behavior, crystallinity, and surface properties are relatively well understood [9,11,12,13,14]. However, translating these insights to pullulan is not straightforward due to its distinct molecular structure. Whereas esterification in cellulose and starch involves both disruption of existing ordered domains and formation of new structural organization, pullulan lacks intrinsic crystallinity [19,20]. Therefore, the influence of alkyl chain length and DS on structure–property relationships is expected to be different and remains insufficiently understood, particularly in nanoscale thin-film systems where nanoscale organization governs surface properties.

The absence of pre-existing crystalline order makes pullulan a particularly suitable platform to isolate the contribution of grafted fatty acid chains to molecular organization and material properties. This facilitates a more direct interpretation of structure–property relationships than is possible with more structurally complex polysaccharides. In addition, pullulan remains highly water-soluble over a broad pH range and exhibits limited conformational variability in solution [17]. These characteristics provide an initially well-defined platform for chemical modification. However, esterification with fatty acids progressively reduces water solubility through the introduction of hydrophobic side chains, resulting in pullulan esters that are insoluble in water and exhibit different solution behavior and surface properties. The ability to control surface wettability, molecular organization, and film morphology through such chemical modification is particularly relevant for coating applications, where interfacial properties play a critical role in performance [21,22,23].

In this work, pullulan was esterified with myristic acid (C14), palmitic acid (C16), and stearic acid (C18), yielding pullulan myristate (PME), pullulan palmitate (PPE), and pullulan stearate (PSE), respectively. The reaction was performed via N,N-carbonyldiimidazole (CDI)-mediated esterification under mild reaction conditions, while varying the DS. Comprehensive characterization was carried out, including Fourier transform infrared spectroscopy (FTIR), proton nuclear magnetic resonance spectroscopy (^1^H-NMR), rheology to determine the viscosity, thermogravimetric analysis (TGA), differential scanning calorimetry (DSC), X-ray diffraction (XRD), water contact angle measurements, scanning electron microscopy (SEM), atomic force microscopy (AFM), and film thickness analysis. This study therefore aims to establish clear structure–property relationships between chemical functionalization with fatty acids of varying alkyl chain length (C14-C18) and the resulting thermal, structural, and surface properties of the materials. Nanoscale films were prepared by spin coating under identical processing conditions for all samples. While processing parameters are known to affect film morphology and uniformity, their optimization was beyond the scope of this work [23,24]. Maintaining constant spin-coating conditions allowed systematic evaluation of the effect of molecular structure on material properties.

## 2. Results

### 2.1. Chemical Characteristics

**Figure 1:**
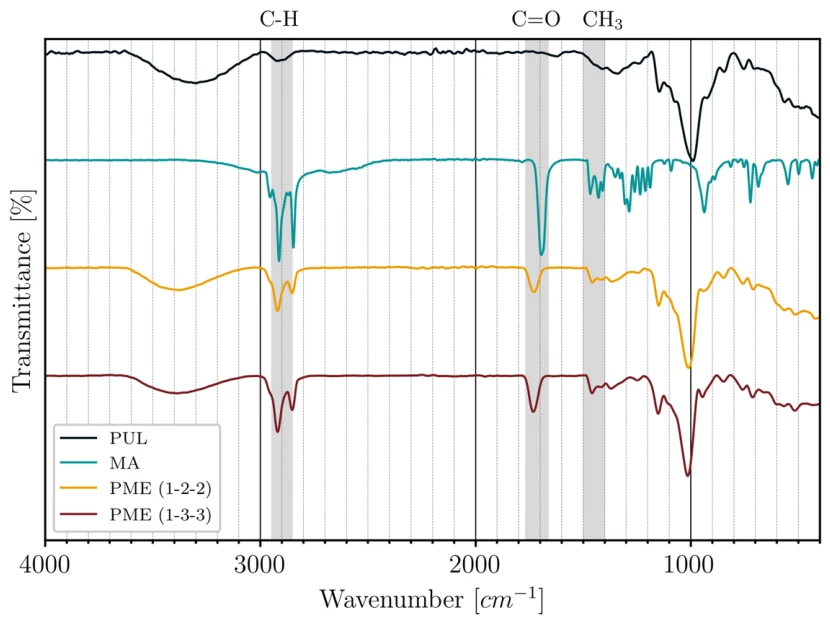
FTIR spectra of pullulan (PUL), myristic acid (MA), and pullulan myristate (PME), synthesized with esterification molar ratios (anhydroglucose unit (AGU)-N,N-carbonyldiimidazole (CDI)-fatty acid) of (1-2-2) and (1-3-3). The spectrum highlights the C-H stretching, C=O stretching, and the characteristic vibrations of methyl groups (CH_3_). A clear shift of the C=O (carbonyl) stretching peak from MA to the corresponding ester C=O peak in PME confirms successful esterification.

To evaluate the efficiency of the esterification reaction, FTIR spectroscopy was utilized. Figure 1 shows the spectra of pullulan, myristic acid (MA), and pullulan myristate (PME) synthesized with molar ratios (anhydroglucose unit (AGU)-CDI-fatty acid) of (1-2-2) and (1-3-3). For pullulan, characteristic peaks corresponding to O-H stretching (3307 cm^−1^), C-H stretching (2914 cm^−1^), and various C-O and glycosidic vibrations are observed, consistent with literature [25,26,27,28,29,30].

The spectra of the fatty acids (MA, PA, SA) are similar and show a broad O-H band (3200–2400 cm^−1^), C-H stretching at 2914 and 2848 cm^−1^, and a carbonyl peak at 1698 cm^−1^ [13,31,32]. Therefore, only the PME spectrum is discussed in detail. After esterification, the pullulan ester spectrum retains the characteristic polysaccharide peaks, while significant changes are observed. The intensity of the O-H stretching band at 3307 cm^−1^ decreases and stronger C-H stretching bands appear at 2920 and 2852 cm^−1^. In addition, a new C=O stretching (ester) peak is observed at 1726 cm^−1^ [31]. Comparison of PME samples shows increased intensity of both C-H stretching and C=O absorption for the (1-3-3) ratio. Similar trends are observed for PPE and PSE (Figure S2) [13,31,32,33].

**Figure 2:**
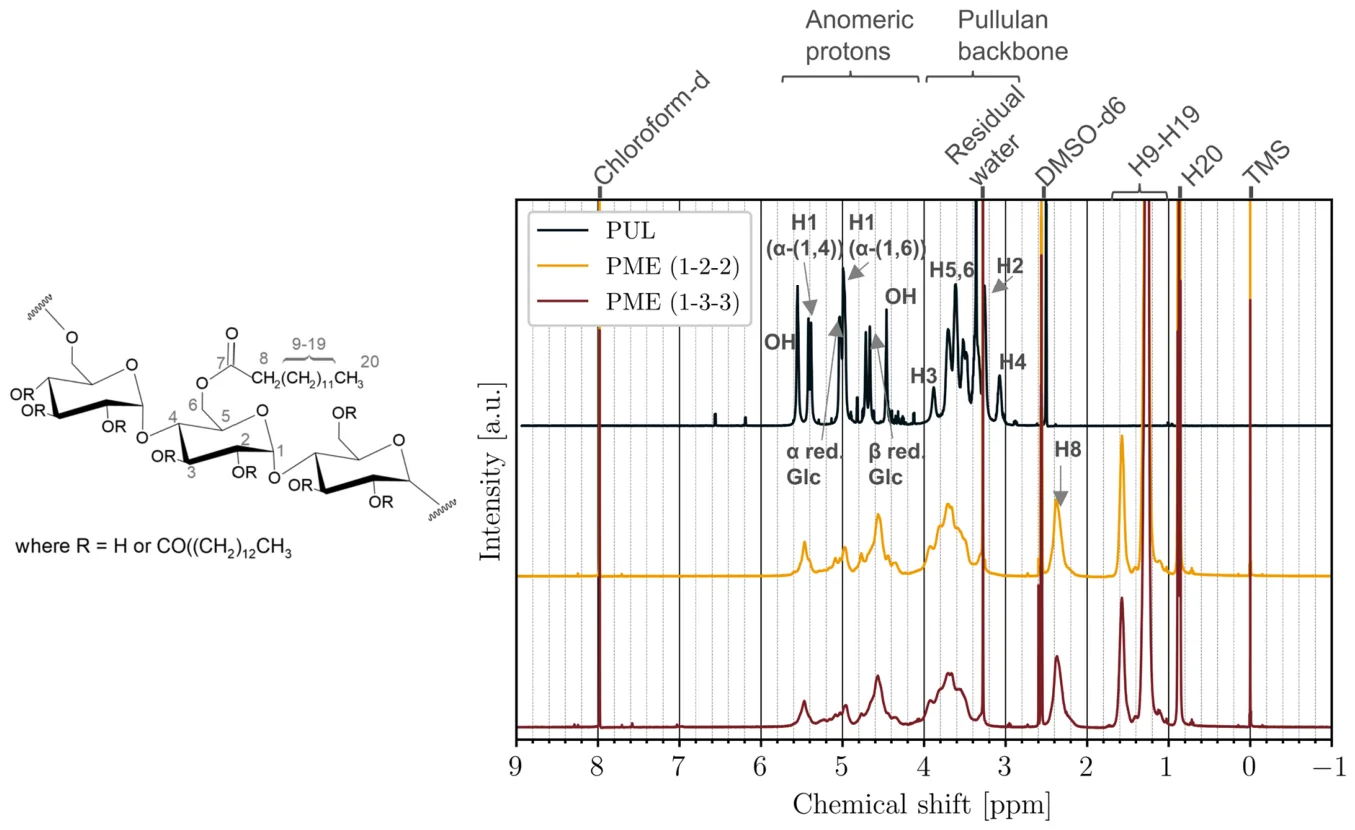
Chemical structure of pullulan myristate (PME) and corresponding ^1^H-NMR spectra of pullulan (PUL) in DMSO-d_6_ compared with pullulan myristate in a chloroform-d/DMSO-d_6_ (60/40, *v*/*v*) solvent mixture, synthesized at esterification molar ratios (AGU-CDI-fatty acid) of (1-2-2) and (1-3-3). The spectrum highlights the characteristic protons of the pullulan backbone and the emergence of aliphatic resonances (H8-H20) associated with the myristoyl moieties, confirming successful esterification. For the PME samples, spectra were normalized to the terminal methyl signal of the fatty acid (~0.87 ppm), facilitating comparison of substitution levels between the two PME samples.

While FTIR provides evidence for the formation of the ester functionalities, ^1^H-NMR was employed to obtain further information on the molecular structure of the modified pullulan esters. The ^1^H-NMR spectra of pullulan in DMSO-d_6_ and of PME (1-2-2) and PME (1-3-3) in a chloroform-d/DMSO-d_6_ (60/40, *v*/*v*) mixture are shown in Figure 2. A mixed solvent system was required to dissolve the pullulan esters, resulting in a shift of the chloroform-d peak from ~7.26 ppm to 7.98 ppm due to hydrogen-bonding interactions with DMSO-d_6_ [34,35], while the DMSO-d_6_ peak remains close to its typical position. For pullulan, signals between 3.00 and 4.00 ppm correspond to the H2-H6 protons of the AGU, while anomeric protons appear between 4.20 and 5.70 ppm. Hydroxyl protons are observed at 4.46 and 5.55 ppm, and H1 protons adjacent to the α-(1,4) and α-(1,6) glycosidic linkages appear at 5.39–5.42 ppm and 4.99 ppm, respectively [30,36].

Following esterification, new signals corresponding to the myristoyl group appear between 0.80 and 2.45 ppm. The terminal methyl group (H20) appears as a triplet at 0.87 ppm, while methylene protons (H9–H19) are observed at 1.26 and 1.57 ppm, and the proton adjacent to the ester linkage at 2.38 ppm [13,31,37]. Comparison of PME samples normalized against the terminal methyl signals shows slightly lower AGU signal intensity for PME (1-3-3) compared to PME (1-2-2). Similar trends are observed for PPE and PSE (Figure S5).

### 2.2. Molecular and Solution Properties

#### 2.2.1. Degree of Substitution

Following structural confirmation, the reaction efficiency was evaluated by determining the DS via elemental analysis, whereas the viscosity and molecular weight were obtained by rheology and GPC, respectively (Table 1). Increasing the molar ratio from (1-2-2) to (1-3-3) results in a consistent increase in DS for all samples, with PME increasing from 0.24 to 0.47, PPE from 0.28 to 0.63, and PSE from 0.35 to 0.77. For both molar ratios, DS increases with increasing alkyl chain length (C14-C18). The highest DS was observed for PSE (1-3-3) (0.77), while the lowest DS was observed for PME (1-2-2) (0.24).

#### 2.2.2. Molecular Weight

Since chemical modification can affect polymer chain conformation and solution behavior, the molecular weight characteristics were determined by GPC/SEC and summarized in Table 1. PPE exhibited a notably lower MW than both commercial pullulan and the other pullulan esters. The weight-average molecular weight ranged from 7.5 × 10^4^ Da for PPE (1-2-2) to 6.4 × 10^5^ Da for PSE (1-3-3), whereas PDI values in the range of 1.67 to 2.90 are obtained, with PPE displaying the lower dispersity among the synthesized esters.

#### 2.2.3. Viscosity

To further evaluate the solution behavior and processability, viscosity measurements were conducted in the coating solvent system. Viscosity values of pullulan fatty ester solutions in chloroform/HFIP (80/20, *v*/*v*) are presented in Table 1. PME showed an increase in viscosity from 2.92 to 3.67 mPa·s when the molar ratio was increased from (1-2-2) to (1-3-3), while PPE increased from 1.94 to 2.16 mPa·s. In contrast, PSE exhibits only a small change (2.91 to 2.97 mPa·s). Overall, PPE consistently displays the lowest viscosity values, whereas PME and PSE display higher and comparable values.

### 2.3. Thermal Characteristics

Besides solution behavior, chemical modification is expected to influence the thermal properties of the materials. Therefore, the thermal behavior was evaluated by TGA and DSC (Table 2, Figure S8). TGA measurements reveal two mass-loss stages for PME and PPE. An initial loss is observed between 90 °C and 142 °C, followed by a main degradation stage between 300 °C and 400 °C [13,31]. In contrast, PSE shows no distinct first degradation step and exhibits a higher degradation onset temperature of 312 °C for PSE (1-2-2) and 327 °C for PSE (1-3-3).

DSC analysis shows no melting transitions for PME and PPE. In contrast, PSE samples exhibit clear melting peaks at 40-42 °C and crystallization temperatures at 21 °C for PSE (1-2-2) and 14 °C for PSE (1-3-3).

### 2.4. Structural Characteristics

**Figure 3:**
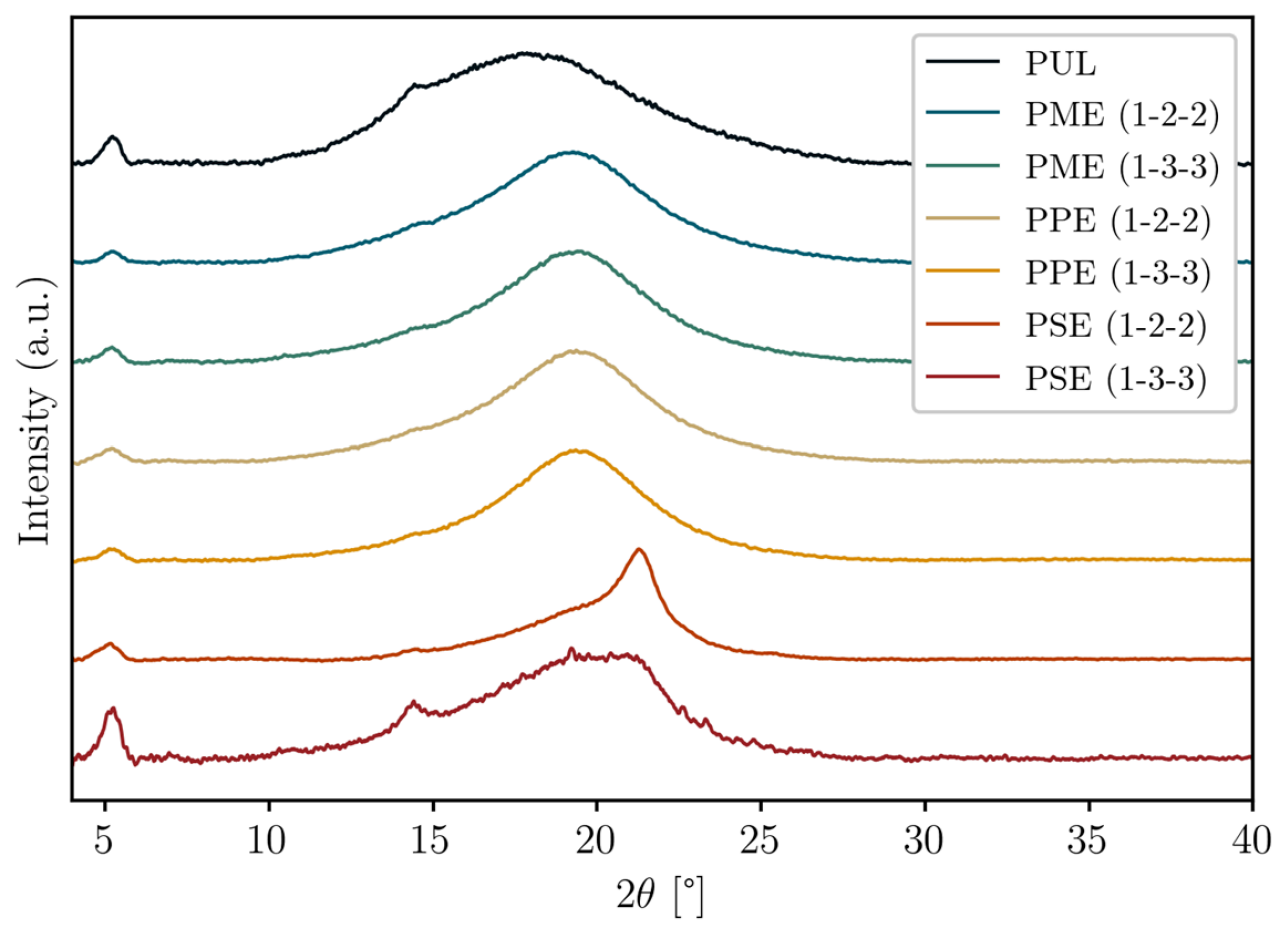
XRD patterns of pullulan (PUL), pullulan myristate (PME), pullulan palmitate (PPE), and pullulan stearate (PSE), synthesized with esterification molar ratios (AGU-CDI-fatty acid) of (1-2-2) and (1-3-3). Amorphous pullulan exhibits a characteristic broad halo. Stearate-modified samples show increased structural ordering compared to the amorphous pullulan backbone.

The thermal transitions observed by DSC suggest differences in molecular organization. Therefore, XRD was used to investigate the structural ordering of the materials. The XRD patterns of pullulan and its fatty acid esters are shown in Figure 3. Pristine pullulan exhibits a broad amorphous halo at approximately 2θ equal to 18°, characteristic of its amorphous nature, along with a smaller feature at 5.2° corresponding to interchain spacing [19,33,38].

Upon esterification, the broad amorphous peak remains present but shows slight narrowing. PME and PPE display relatively broad diffraction patterns, whereas PSE shows more distinct diffraction features. In particular, PSE (1-2-2) exhibits a diffraction peak at 21.3°. Differences in peak intensity and broadness were observed between PSE (1-2-2) and PSE (1-3-3).

### 2.5. Surface Characteristics

#### 2.5.1. Surface Morphology (SEM)

Following characterization of the molecular and structural properties, the surface morphology of the spin-coated films was evaluated by SEM at magnifications of ×100, ×10,000, and ×30,000, focusing on the coating edge, where local variations are most pronounced (Figure S9). At low magnification, all coatings appear continuous and smooth, with only minor defects or faint lines, likely originating from spin-coating and controlled solvent evaporation. Along the coating edge, a thicker ridge is observed together with occasional wavy edge features.

Higher magnification reveals that PSE samples exhibit faint flow-aligned striations, corresponding to small variations in film thickness. However, no pronounced striations are observed overall, confirming that the processing conditions yield relatively uniform coatings (Figure S3). At the highest magnification, all samples display fine crack networks. Cracking is more pronounced for samples with longer alkyl chains and for PME (1-2-2).

#### 2.5.2. Surface Topography and Film Thickness (AFM)

In addition to the SEM observations and to obtain quantitative information on the surface topography, AFM analysis was performed (Figure S10), with results summarized in Table 3. All samples exhibit extremely smooth nanoscale topographies, with mean roughness (S_a_) ranging from 0.34 to 0.69 nm and RMS roughness (S_q_) from 0.43 to 1.01 nm.

Among the samples, PPE (1-2-2) shows the highest roughness (S_a_ = 0.69 ± 0.08 nm; S_q_ = 1.01 ± 0.21 nm). In contrast, PME samples exhibit the lowest roughness values (~0.34 nm). PSE samples combine relatively low roughness with a more homogeneous height distribution. Nanoscale film thicknesses are observed in the range of approximately 110 to 150 nm, with a general decrease observed at higher DS.

#### 2.5.3. Surface Wettability (Contact Angle)

Since surface morphology and chemistry influence wettability, the surface hydrophobicity of the coatings was assessed through water contact angle measurements. The water contact angle was measured at 20 °C and the results are summarized in Table 3. Contact angles ranged from 85° to 105°. In general, contact angles increased with increasing alkyl chain length and with increasing molar ratio. The lowest contact angle was observed for PME (1-2-2) (85 ± 7°), while the highest value was measured for PSE (1-3-3) (105 ± 2°).

### 2.6. Biological Characteristics

The biological performance of the pullulan ester coatings was evaluated through cytotoxicity, cell adhesion, and antimicrobial experiments to determine how the observed surface properties influence cellular and bacterial interactions.

#### 2.6.1. Cytotoxicity Studies

**Figure 4:**
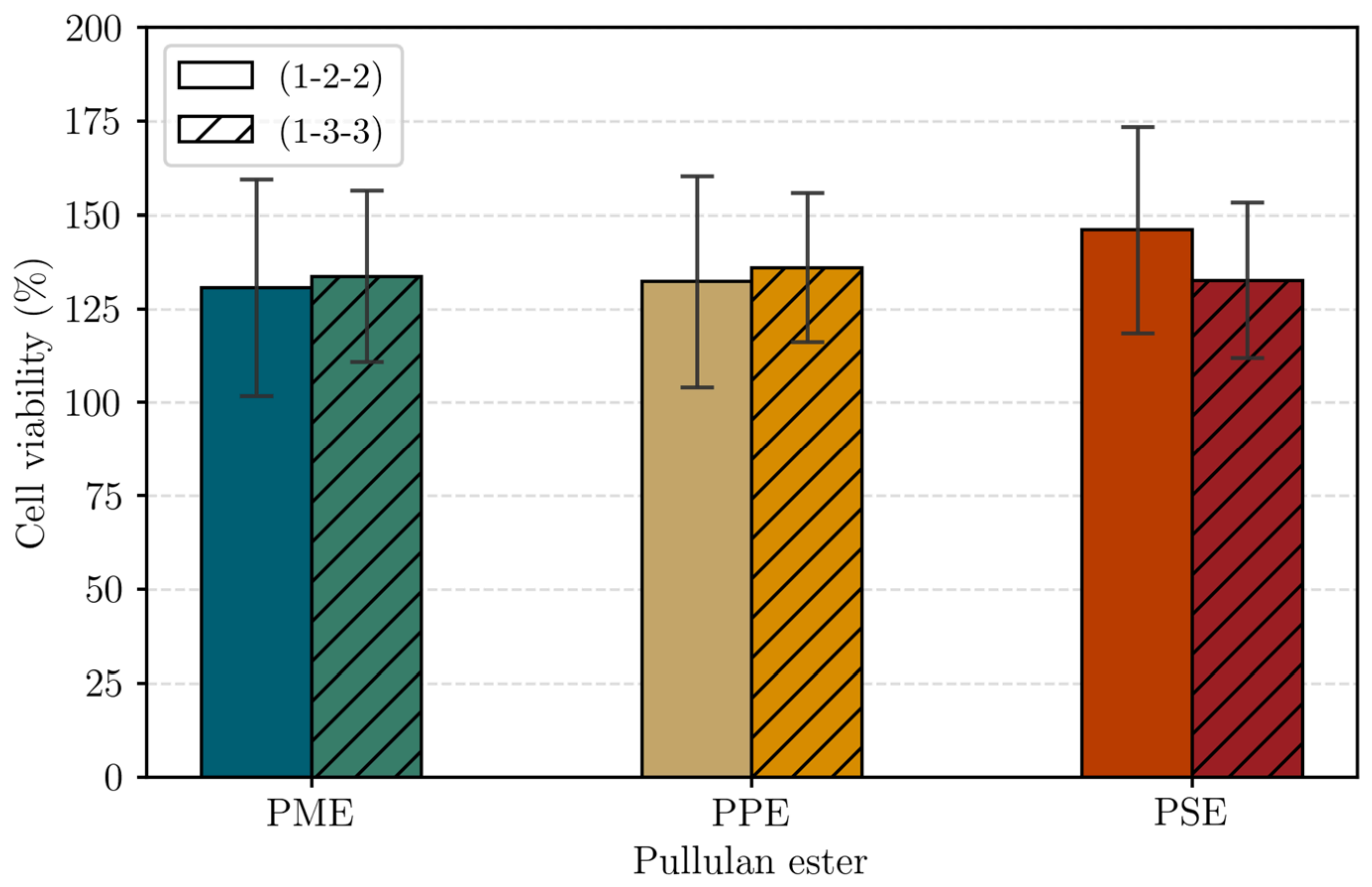
Cell viability of NIH3T3 fibroblasts following 24 h exposure to extract medium derived from pullulan myristate (PME), pullulan palmitate (PPE), and pullulan stearate (PSE) coatings at esterification molar ratios (AGU-CDI-fatty acid) of (1-2-2) and (1-3-3), assessed using the MTT assay. Fresh DMEM served as the negative control (100% viability) and 20% DMSO as the positive control. All samples show high viability, confirming non-cytotoxic behavior. Values are expressed as percentage viability relative to the negative control.

The in vitro cytocompatibility of NIH3T3 fibroblasts on pullulan-fatty acid ester surfaces was assessed according to ISO 10993-5, which describes the biological evaluation of medical devices. The results of the MTT assay, illustrated in Figure 4, show that all tested substrates exhibit high cell viability and no cytotoxic effects, with average cell viability values ranging from 130.7% for PME (1-2-2) to 146.1% for PSE (1-2-2) relative to the negative control. No significant differences are observed between the different samples.

#### 2.6.2. Cell Adhesion Studies

**Figure 5:**
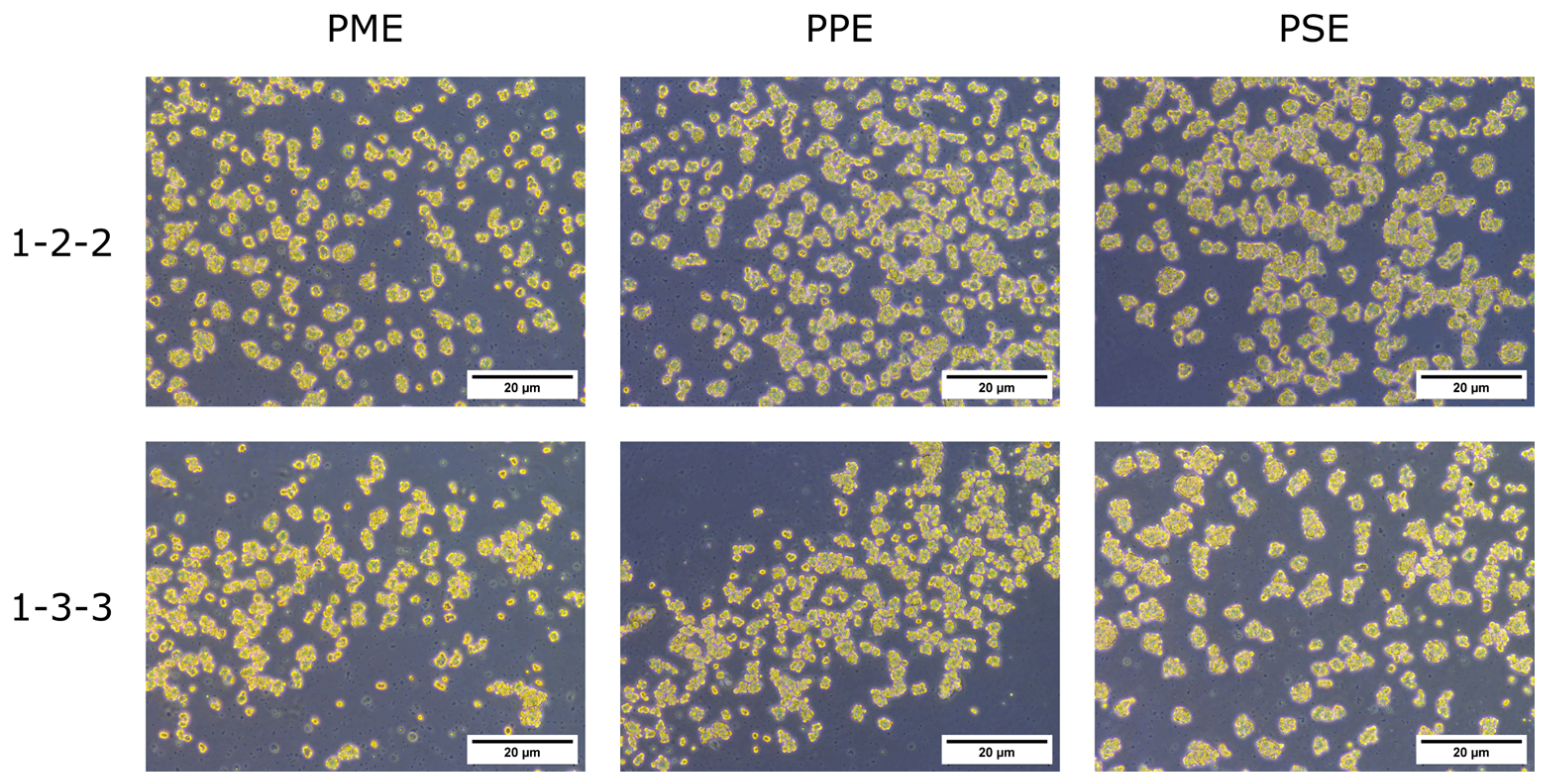
Bright-field microscopy images of bovine aortic endothelial cells (BAEC/BAOEC) cultured for 24 h on pullulan myristate (PME), pullulan palmitate (PPE), and pullulan stearate (PSE) at esterification molar ratios (AGU-CDI-fatty acid) of (1-2-2) and (1-3-3). Cells across all modified surfaces exhibit a rounded morphology with absent cell spreading, indicating poor cell-surface interaction and lack of endothelial cell adhesion after 24 h of culture.

Inverted microscopy images (Figure 5) show the absence of endothelial cell (BAEC/BAOEC) adhesion on all pullulan fatty ester coatings after 24 h of incubation. The cells remain spherical and non-spread rather than flattened, indicating low cell adhesion.

#### 2.6.3. Antimicrobial Activity

**Figure 6:**
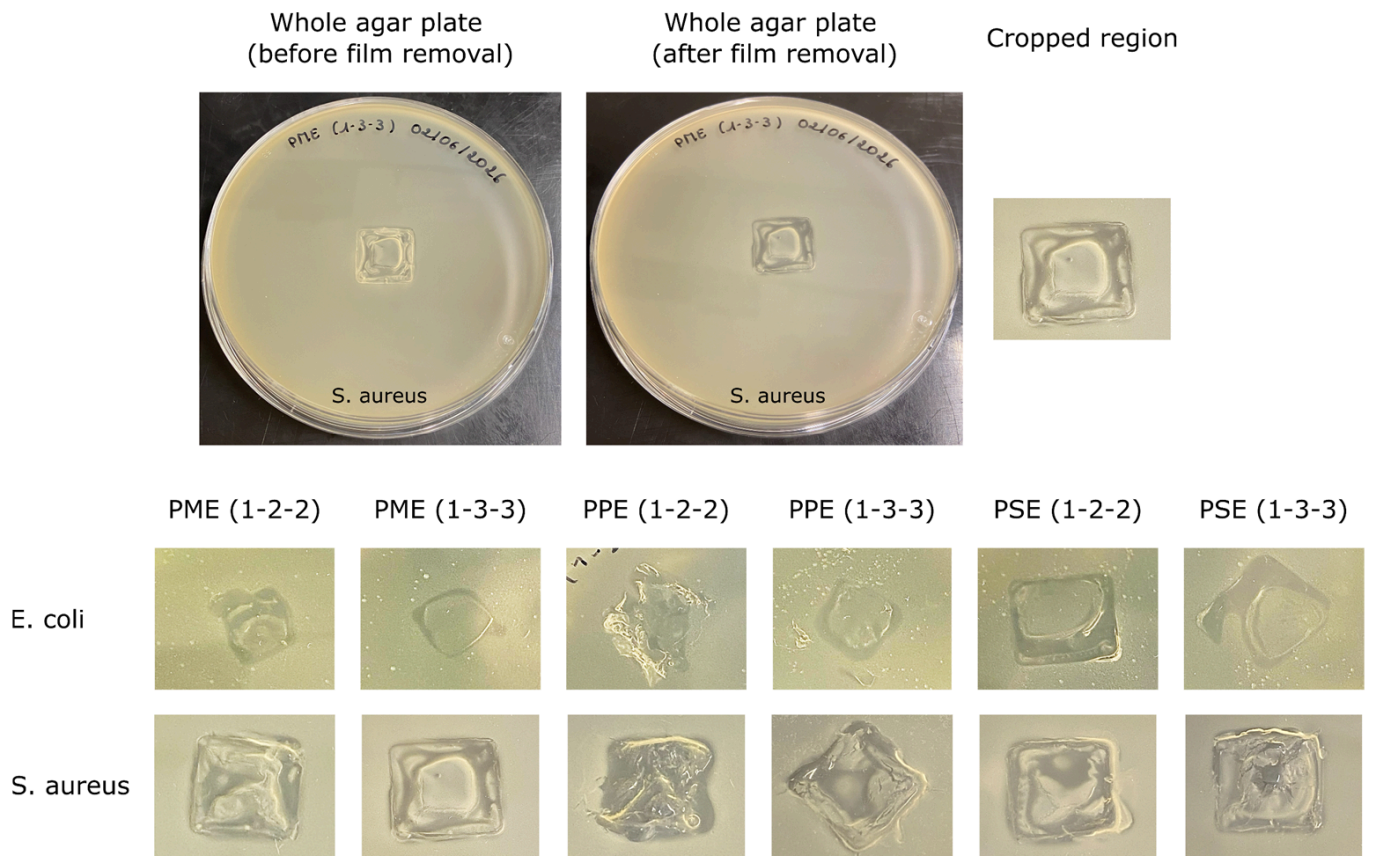
Antimicrobial activity of pullulan myristate (PME), pullulan palmitate (PPE), and pullulan stearate (PSE) films assessed against *Escherichia coli* and *Staphylococcus aureus* using an agar diffusion method following overnight incubation. The absence of clear inhibition zones combined with a lack of bacterial growth directly beneath the film suggests a localized contact-dependent effect without the leaching of antimicrobial agents into the surrounding agar medium.

An agar diffusion test was performed to evaluate antimicrobial activity against Gram-negative *E. coli* and Gram-positive *S. aureus* (Figure 6). No inhibition zones were observed, indicating that no antimicrobial agents are released from the coatings into the surrounding medium.

However, after removal of the samples, the contact area on the agar region that had been in direct contact with the films remains visibly free of bacterial growth, whereas bacterial growth was observed in the surrounding areas. In addition, no bacterial layer was detected on the removed film surfaces.

## 3. Discussion

The results of the chemical, physical, and biological characterization provide insight into how alkyl chain length and degree of substitution influence the molecular, thermal, structural, surface, and biological properties of pullulan fatty esters. The relationships between these parameters and the resulting material performance are discussed in the following sections.

### 3.1. Esterification of Pullulan with Fatty Acids

FTIR analysis supports successful esterification of pullulan. The appearance of a new carbonyl peak at 1726 cm^−1^, corresponding to ester C=O stretching, together with the disappearance of the fatty acid carbonyl signal at 1698 cm^−1^, indicates the formation of ester linkages. Furthermore, the decrease in O-H intensity (3307 cm^−1^) indicates the consumption of hydroxyl groups during the reaction, while the increased intensity of the aliphatic C-H stretching bands at 2920 and 2852 cm^−1^ is consistent with the incorporation of fatty acid side chains [31]. Moreover, the higher intensity of carbonyl and aliphatic bands for the (1-3-3) formulations is consistent with a higher DS, in agreement with the elemental analysis results [13,31,32,33].

The FTIR observations are further supported by the ^1^H-NMR results, suggesting successful grafting of fatty acid chains onto the pullulan backbone. While the AGU and anomeric signals of pullulan remain visible, new resonances in the aliphatic region (0.80–2.45 ppm) appear, indicating modification and preservation of the backbone structure [13,31,37]. Furthermore, the reduced relative intensity of the AGU signals for the (1-3-3) formulations suggests a higher extent of substitution, which is consistent with the DS values measured by elemental analysis.

Beyond confirming successful modification, the elemental analysis results provide insight into the efficiency of the esterification reaction. The measured DS values demonstrate that the DS depends on reagent concentration, with higher molar ratios promoting more efficient esterification of the hydroxyl groups on the pullulan backbone. In addition, DS increased with alkyl chain length, suggesting that longer fatty acids favor esterification, potentially due to differences in solubility, reaction environment, or hydrophobic interactions during the reaction.

Although successful esterification was achieved, the obtained DS values (0.24–0.77) remain lower than those of fully substituted cellulose derivatives (DS = 1.7–2.8) [9,12]. In cellulose systems, high DS values are typically achieved under homogeneous conditions using strong solvent systems (LiCl/DMAc). Starch esters synthesized in DMSO can reach DS values above 2, while dextran esters generally exhibit intermediate to high DS values (~0.94–3) depending on conditions, with their branched structure influencing hydroxyl accessibility and potentially leading to heterogeneous substitution patterns [11,13,14,31].

For pullulan, the linear and amorphous α-(1,4)- and α-(1,6)-linked backbone ensures relatively uniform initial accessibility of hydroxyl groups. However, as the reaction proceeds, this uniformity is restricted by solubility limitations. At higher CDI and fatty acid concentrations, especially for longer chains (C14-C18), the modified pullulan becomes increasingly more apolar and insoluble in DMSO, leading to precipitation and reduced accessibility of remaining hydroxyl groups. In addition, increasing substitution may introduce steric hindrance around unreacted hydroxyl groups, while differences in the accessibility and reactivity of primary and secondary hydroxyl groups may further influence the esterification efficiency. Nevertheless, precipitation of the increasingly hydrophobic products is considered the primary factor limiting further substitution under the investigated reaction conditions. A similar limitation has been reported for starch esters, where phase separation restricts further substitution [14]. Despite these limitations, reproducible DS values ranging from 0.24 to 0.77 were obtained, demonstrating that the CDI-mediated esterification approach enables systematic control of pullulan functionalization by varying the reagent ratio and fatty acid chain length.

### 3.2. Influence of Alkyl Chain Length and DS on Polymer Conformation and Solution Behavior

These variations in DS and alkyl chain length directly alter intermolecular interactions, dictating the overall polymer conformation and solution behavior. Consequently, the MW values obtained by GPC/SEC represent apparent molecular weights, as they depend on the hydrodynamic volume of the polymer coils rather than the absolute mass [39]. Changes in DS and alkyl chain length affect hydrogen bonding and intermolecular interactions, thereby altering chain conformation and solubility [14]. As a result, MW may increase or decrease depending on whether chain expansion or compaction dominates.

In the present study, PPE exhibits a notably lower apparent MW compared to both commercial pullulan and the other pullulan esters (Table 1). This behavior may be attributed to a more compact chain conformation in THF, driven by intramolecular hydrophobic interactions. However, alternative explanations related to polymer–solvent interactions cannot be excluded. The combination of intermediate alkyl chain length (C16) and modest DS values may reflect differences in hydrodynamic volume arising from altered polymer–solvent interactions. The PDI values further show that, although PPE exhibits a somewhat lower dispersity than PME and PSE, all samples display relatively broad molecular weight distributions (PDI~1.7–2.9). This may reflect a notable degree of heterogeneity introduced during esterification, potentially arising from localized phase separation and precipitation during the reaction [14]. These observations further emphasize that the measured MW reflects solution behavior rather than a true decrease in polymer mass. The influence of alkyl chain length further contributes to these observations. Longer chains (C18) enhance hydrophobic interaction, but may also promote side-chain ordering, limiting chain contraction.

It should also be noted that GPC measurements were performed in THF, where differences in solubility and polymer–solvent interactions may further enhance chain conformation, particularly for pullulan esters with controlled hydrophobicity. Consequently, the proposed chain-compaction mechanism should be regarded as a plausible interpretation rather than definitive proof. Additional GPC measurements in alternative solvent systems would be valuable for further verification.

Similar trends are reported for other polysaccharide fatty esters. In cellulose systems (C10–C16), incorporation of hydrophobic side chains results in mass increases of 135–289% for DS values between 1.7 and 3 [12]. In starch laurates, MW increases from 1.90 × 10^6^ Da to 4.92—6.36 × 10^6^ Da due to disruption of amylopectin aggregates at elevated temperature (≥100 °C), while minor degradation at higher DS is offset by the added mass [14,40]. Dextran esters (C2–C12) exhibit weight-average MW ranging from 1.5 × 10^5^ to 3.2 × 10^5^ Da at complete substitution (DS ≈ 3.0) [11]. These systems similarly demonstrate that apparent MW reflects a balance between chemical modification, chain conformation, and intermolecular interactions.

While GPC/SEC tracks conformational changes through hydrodynamic volume, viscosity data evaluate solution behavior under conditions tailored for spin coating, as it directly influences film formation during spin coating [23]. In contrast to THF used for GPC analysis, this chloroform/HFIP solvent provides a more suitable environment for dissolving hydrophobic pullulan esters and may reduce chain compaction effects observed in molecular weight measurements.

The viscosity trends are consistent with the molecular weight results. In particular, PPE exhibits the lowest viscosity, suggesting a reduced hydrodynamic volume in solution. This observation is consistent with a more compact chain conformation for PPE compared to PME and PSE, explaining the lower apparent molecular weights.

Overall, viscosity is only moderately affected by DS but shows a clearer dependence on alkyl chain length, reflecting the balance between reduced hydrogen bonding, hydrophobic interactions, and chain entanglement [14]. The relatively small viscosity differences may also be related to the low polymer concentration and the short measurement time, which limit the development of pronounced chain entanglement and steady-state flow behavior. The overall stability of the values further indicates that no major degradation occurs and that differences in apparent MW may originate from conformational effects rather than changes in polymer backbone length. Limited data on viscosity are available for other polysaccharide fatty esters, as most studies emphasize MW, DS, and thermal properties. In addition, direct comparison between studies is complicated by differences in polymer concentration, solvent system, temperature, and measurement methodology. Together, the molecular weight and viscosity data show that alkyl chain length and DS influence solution behavior primarily by altering the polymer conformation and intermolecular interactions.

### 3.3. Influence of Alkyl Chain Length and DS on Thermal Behavior and Structural Ordering

The observed changes in solution behavior are expected to influence molecular packing and intermolecular interactions of the solid pullulan fatty esters. Therefore, the effects of alkyl chain length and DS on the thermal behavior and structural ordering were examined. Changes in molecular structure, particularly DS and alkyl chain length, influence both the thermal stability and structural organization of the materials by modifying the balance between hydrogen bonding and hydrophobic interactions.

The thermal results suggest that alkyl chain length is the dominant factor controlling thermal behavior. A low temperature mass-loss step is observed for several formulations between approximately 90 °C and 142 °C. However, because this feature is less pronounced or absent for some samples, its origin cannot be assigned unambiguously. Therefore, this mass loss may originate from a combination of residual moisture evaporation and/or degradation of less thermally stable fatty acid-associated domains. However, the absence of a distinct low temperature degradation step for PSE, together with its higher degradation onset temperature, suggests improved thermal stability with increasing alkyl chain length.

The DSC results further highlight the influence of alkyl chain length. While PME and PPE exhibit no melting transition, PSE displays clear melting and crystallization peaks, suggesting that C18 side chains are sufficiently long to promote side-chain crystallization and the formation of ordered domains, while shorter chains (C14–C16) lack sufficient length to induce crystallinity [9]. Consequently, thermal transitions are primarily driven by alkyl chain length rather than DS [12]. This behavior is consistent with other polysaccharide fatty esters. Cellulose esters (C8–C18) show melting transitions typically between 43 and 59 °C in the first heating cycle, with longer chains leading to higher melting temperatures and increased crystal thickness after repeating the heating cycle [9]. In dextran systems, palmitate derivatives exhibit melting temperatures of 39–45 °C for DS values between 0.94 and 1.89 and remain stable up to ~195 °C [13,31]. In pullulan esters, melting is observed only for stearate-modified samples (C18), indicating that a critical chain length is required for pullulan to induce side-chain crystallization. Unlike cellulose and starch, where both backbone and side-chain ordering contribute to thermal transitions, the amorphous nature of pullulan ensures that these transitions originate solely from side-chain interactions, enabling clear structure–property relationships.

The thermal transitions observed by DSC, particularly for stearate-modified samples, suggest the formation of ordered domains within the material. To further investigate this structural organization, XRD analysis was performed. Quantitative crystallinity determination was not performed because the XRD patterns are dominated by broad amorphous scattering and only limited crystalline contributions are observed, making the calculated values highly dependent on the chosen fitting procedure. Therefore, the XRD data were primarily interpreted qualitatively to evaluate relative changes in structural organization between the samples. The retention of the peak near 5.2° indicates that the pullulan backbone remains largely intact after esterification. Additionally, the narrowing of the broad amorphous halo reflects increased structural organization relative to unmodified pullulan.

The influence of alkyl chain length is particularly evident. The relatively short alkyl chains (C14–C16) of PME and PPE appear insufficient to promote extensive crystallization, while the longer C18 chains of PSE promote the formation of more locally ordered domains through enhanced van der Waals interactions, resulting in sharper diffraction features.

This effect is most pronounced for PSE (C18), where the longer side chains enable the formation of locally ordered side-chain domains, consistent with the melting transitions observed by DSC (Table 2) [9,12,41]. For PSE (1-2-2), a distinct peak at 21.3° is observed, indicating ordered side-chain packing even at moderate DS [33]. For PSE (1-3-3), however, this diffraction feature appears less pronounced, despite the larger melting transition for PSE (1-3-3). This may indicate that local structural ordering reflected by a specific diffraction peak does not necessarily correlate with the total ordered phase contributing to the melting enthalpy. Therefore, the XRD and DSC measurements yield complementary perspectives on the structural ordering of these stearate-modified pullulan derivatives. In addition, higher substitution levels may increase steric hindrance from bulky side groups that partially disrupt regular packing, leading to broader or less intense peaks due to phase segregation and imperfect lamellar organization [42,43,44].

Similar trends have been reported for other polysaccharide fatty esters. In cellulose esters, esterification disrupts hydrogen bonding to form a layered structure. For alkyl chains larger than C14, these side chains can crystallize, increasing the overall crystallinity and melting temperature [9]. Dextran esters remain largely amorphous for shorter chains but can exhibit crystalline features for long-chain derivatives such as palmitates [11,13,31]. In contrast, the intrinsic amorphous nature of pullulan ensures that any increase in ordering arises solely from side-chain packing. This allows direct correlation between alkyl chain length and structural organization, explaining the emergence of more defined diffraction peaks for stearate-modified samples. Overall, these findings demonstrate that alkyl chain length is the primary factor dictating thermal behavior, side-chain crystallization, and structural ordering, whereas DS primarily tunes the degree of chain packing and ordering without altering the overall trend.

### 3.4. Structure–Property Relationships in Thin-Film Coatings

Differences in molecular organization and solution behavior ultimately influence thin-film formation during spin coating. Consequently, their effects on the coating morphology, topography, and wettability are discussed. SEM observations indicate that the selected processing conditions produce continuous and homogeneous thin films. The use of a heating plate at 60 °C, close to the boiling points of chloroform and HFIP, enables gradual solvent removal, resulting in uniform thin films. In addition, the thicker ridge at the coating edge is characteristic of the edge-bead effect, where centrifugal forces drive the solution outward, while viscous and surface tension retain a local excess [45]. Subsequent heating reduces this ridge, occasionally leading to wavy edge features.

The faint flow-aligned striations observed for PSE at higher magnification are likely associated with flow and solvent evaporation instabilities during film formation, which can induce minor thickness fluctuations influenced by viscosity, solubility, and solvent volatility [46,47]. The origin of the crack networks is less certain. These features may result from electron beam-induced degradation of the biopolymer matrix [48]. Alternatively, they may result from residual stresses generated during rapid solvent evaporation and film solidification. As the polymer transitions from a mobile solution to a glassy state, shrinkage is constrained by adhesion to the substrate, leading to tensile stresses that eventually result in crack formation [49,50]. The more pronounced cracking observed for samples with longer alkyl chains suggests that reduced chain mobility may contribute to stress accumulation, while the behavior of PME (1-2-2) further indicates that the solution viscosity and diffusion during film thinning additionally influence stress development [22,49]. This is in line with studies demonstrating that viscosity and solubility affect film thickness, homogeneity, and stress formation during spin coating [51].

In contrast to the SEM observations, AFM provides quantitative information on the nanoscale topography of the coatings. These results confirm that all coatings remain highly smooth at the nanoscale, strongly indicating that the crack networks observed previously under SEM are artifacts of the high-vacuum analysis or metal deposition rather than intrinsic features of the film [48]. Differences in roughness between the samples provide further insight into the molecular organization. PPE (1-2-2) exhibits the highest roughness, consistent with a more heterogeneous surface, whereas PME showed the most uniform topography. PSE samples combine relatively low roughness with a more homogeneous height distribution, suggesting improved packing. This behavior can be linked to the longer alkyl chains (C18), which promote more regular side-chain organization, in agreement with the thermal and XRD results.

In addition to the surface topography, coating thickness measurements offer further insight into the film formation process. A general decrease in film thickness is observed at higher DS. Although viscosity influences the film formation, the relatively small variations in viscosity (~2–4 mPa·s) indicate that film thickness is controlled by a combination of factors, including solvent evaporation, polymer-substrate interactions, and chain mobility, rather than viscosity alone. Increasing DS reduces intermolecular hydrogen bonding and enhances the hydrophobic character of the polymer chains, improving compatibility with the chloroform-rich solvent phase. This promotes more uniform chain dispersion during spin coating and facilitates efficient packing, resulting in denser films with slightly reduced thickness at higher DS.

Compared to other polysaccharide fatty ester coatings, limited data are available. For example, patterned, spin-coated dextran esters have been reported, with thicknesses of approximately 1 µm and surface roughness values of ~6.97 nm (S_a_) and ~9.26 nm (S_q_) [13]. These values are significantly higher than those observed for pullulan esters in this study, since nanoscale patterning greatly affects the roughness parameters. The combination of nanoscale thickness and sub-nanometer roughness demonstrates that pullulan fatty esters can be processed into uniform nanostructured coating layers.

The observed differences in morphology, topography, and molecular organization are expected to influence the wettability of the coatings. Increasing alkyl chain length leads to higher contact angles, reflecting the increased hydrophobicity of longer fatty acid side chains. Similarly, contact angles increase with molar ratio, which correlates with the higher DS. A higher DS results in a greater number of hydrophobic groups at the surface, yielding moderate contact angles between 85° and 105° rather than superhydrophobic behavior [13,52,53].

Despite clear overall trends, relatively large standard deviations are observed, which may be attributed to small variations in surface morphology. Although SEM shows largely smooth coatings, minor thickness variations and fine crack networks are present at higher magnification, while AFM confirms a low roughness. These features can locally affect wetting behavior by influencing droplet spreading, resulting in variability in the measured contact angles. In addition, the relatively low DS of the pullulan esters compared to some other polysaccharide fatty esters may contribute to this variability. Due to incomplete surface coverage by hydrophobic side chains, both hydrophilic pullulan backbone segments and hydrophobic alkyl chains can be exposed at the interface. As a result, the deposited water droplet may locally interact with regions of differing polarity, further contributing to fluctuations in the measured contact angle.

The wettability behavior is consistent with trends reported for other polysaccharide fatty esters. Cellulose esters typically exhibit contact angles between ~93° and 107°, with hydrophobicity increasing with chain length and DS [9]. Starch generally shows contact angles greater than 95°, whereas higher values (~112°) have been reported for patterned dextran palmitate films [13,40]. For pullulan esters, the intermediate contact angles (85–105°) indicate moderately hydrophobic surfaces, resulting from the balance between hydrophobic side chains and the hydrophilic polysaccharide backbone. Furthermore, because the present work does not focus on reaction optimization, the maximum degree of substitution and corresponding upper limit of the surface hydrophobicity achievable for pullulan fatty esters remain to be determined. Given the nanoscale thickness and smoothness of the coatings, the observed wettability differences are primarily attributed to differences in surface chemistry rather than microscale roughness effects. Overall, coating surface properties are driven primarily by surface chemistry, where alkyl chain length and DS dictate the balance between hydrophilic pullulan backbone segments and hydrophobic fatty acid side chains.

### 3.5. Biological Implications of Surface Properties

Since biological interactions occur at the coating interface, variations in surface morphology and topography will likely influence cell-material responses. The biological performance of these materials is therefore evaluated through cytotoxicity, cell adhesion, and antimicrobial testing. The high cell viability observed for all samples indicates that the chemical modification of pullulan with fatty acids does not introduce cytotoxic effects under the tested conditions. Similar findings have been reported for other polysaccharide fatty esters, where the biocompatibility is largely dictated by the polysaccharide backbone [13]. Minor variations in cell viability between the samples do not show a clear dependence on alkyl chain length or DS, suggesting that these parameters have a limited influence on cytocompatibility within the investigated range. These results confirm that the modified pullulan systems retain the inherent biocompatibility of the native polysaccharide, although further studies are required to assess long-term biological responses.

While the coatings remain cytocompatible, they strongly limit endothelial cell adhesion. This behavior likely arises from a combination of factors rather than a single dominant mechanism. First, the polysaccharide backbone lacks specific biological adhesion sites, preventing integrin-mediated attachment [54]. In addition, the surface chemistry, characterized by a combination of hydrophobic fatty acid moieties and residual hydrophilic groups, may hinder stable protein adsorption required for cell adhesion and spreading. Among these factors, reduced protein adsorption may contribute to bio-inert response, as initial protein adsorption typically mediates subsequent cell attachment and spreading.

Surface morphology may further contribute to the observed bio-inert response. Furthermore, the relatively smooth surface morphology and intermediate wettability (~85–105°) reduce opportunities for mechanical anchoring and spreading of cells [55,56]. Comparable behavior has been reported for other polysaccharide-based systems. For example, dextran palmitate coatings can support endothelial cell growth under certain conditions, while surface patterning or smooth regions can significantly affect adhesion and cell organization [13]. The formation of a hydrated interfacial layer due to the polysaccharide backbone cannot be excluded and may contribute to reduced cell adhesion. However, as no direct protein adsorption measurements were performed, neither the role of protein adsorption nor that of a hydrated interfacial layer can be conclusively confirmed. Overall, these results indicate that the pullulan ester coatings exhibit a bio-inert surface response under the tested conditions. These findings suggest that surface chemistry plays a dominant role over topography in determining both wettability and biological response.

The bacterial response further supports the importance of surface-mediated interactions. The absence of inhibition zones indicates that no antimicrobial compounds are released from the coatings into the surrounding medium. However, the absence of bacterial growth at the contact area suggests a localized surface-mediated inhibition of bacterial growth. Similar contact-active behavior has been reported for other polysaccharide-based systems, where surface chemistry and wettability influence bacterial adhesion and proliferation [55]. Although the exact mechanism remains unclear, the combined cell adhesion and antimicrobial tests indicate that the pullulan ester coatings exhibit a bio-inert surface character while maintaining excellent cytocompatibility. Collectively, these findings demonstrate that fatty acid-modified pullulan can provide bio-inert interfaces that resist both cellular and bacterial interactions without introducing cytotoxic effects.

### 3.6. Limitations and Future Directions

While these findings establish clear structure–property relationships, evaluating the broader applicability of these materials requires further investigation. The present study focused on structure–property relationships of fatty-acid modified pullulan, employing identical reaction and coating conditions. Therefore, reaction optimization of parameters such as temperature, reaction time, and solvent composition was beyond the scope of this work. Additionally, the molecular weight measurements were performed using different solvent systems for commercial pullulan and the pullulan esters, while alternative solvents were required for GPC, NMR, and rheological characterization. Similarly, the thermal and structural characterization mainly focuses on identifying side-chain ordering effects, while low-temperature DSC measurements may provide additional insight into glass-transition behavior and segmental chain mobility, and variable-temperature XRD studies may be used to directly monitor crystallization and melting processes.

From an application perspective, future studies may focus on fluorine-free coating solvents, optimization of the spin coating conditions for each pullulan fatty ester derivative, and long-term stability experiments under physiological conditions. In addition, characterization of the coating adhesion, mechanical durability, and wear resistance would provide valuable information regarding the practical implementation of pullulan ester nanocoatings. Moreover, direct protein adsorption measurements may provide insights into the underlying mechanisms of the observed bio-inert behavior. However, as only preliminary in vitro experiments were conducted, further studies are required to clarify the underlying mechanisms and to quantitatively compare these materials with other polysaccharide-based coatings. Despite these limitations, the present results provide a comprehensive framework for understanding how alkyl chain length and degree of substitution control the structural, surface, and biological properties of pullulan fatty esters and support their future development as functional nanocoating materials.

## 4. Materials and Methods

### 4.1. Materials

Pullulan (MW_w_ 1.6 × 10^5^ Da) was acquired from Tokyo Chemical Industry (TCI Europe NV). Myristic acid, stearic acid, hydrochloric acid (37%), phenolphthalein (1% in water), tetrahydrofuran (THF), DPBS (lacking calcium and magnesium salts), Dulbecco’s Modified Eagle Medium (DMEM with high glucose and pyruvate), and TrypLE Express Enzyme (1X) (phenol red-free) with EDTA were obtained from Fisher Scientific BV (Breda, The Netherlands). N,N-Carbonyldiimidazole (CDI) was procured from TCI Europe NV. Palmitic acid and dimethyl sulfoxide-d_6_ (DMSO-d_6_) were purchased from Acros Organics BV (Geel, Belgium). Dimethyl sulfoxide, 1,1,1,3,3,3-Hexafluoro-2-propanol (HFIP), Thiazolyl Blue Tetrazolium Bromide (MTT powder), and chloroform-d (containing 0.03 vol% TMS) were sourced from Merck Life Science BV (Hoeilaart, Belgium). Ethanol (99%), sodium hydroxide, chloroform, and agar were ordered from VWR International BV (Orlyplein, Amsterdam). Lysogeny broth (LB X964.1) was purchased from Carl ROTH (Karlsruhe, Germany). For the in vitro experiments, fibroblasts (NIH3T3), endothelial cells (BAEC/BAOEC), Escherichia coli (*E. coli*), and Staphylococcus aureus (*S. aureus*) were utilized.

### 4.2. Esterification of Pullulan with Fatty Acids

A two-step esterification of pullulan with fatty acids was performed using CDI activation (Figure S1). The procedure employed varying molar ratios of anhydroglucose units of pullulan (AGU) to CDI and fatty acid (1-2-2 and 1-3-3). To prevent excess CDI, equimolar quantities of CDI and fatty acid were used. The selected reaction conditions (70 °C, 24 h) were based on preliminary experiments and previous studies on polysaccharide esterification, and were kept constant to enable direct comparison of the influence of alkyl chain length and reagent ratio on the material properties. Initially, the fatty acid was dissolved in 50 mL of DMSO at 70 °C, followed by addition of CDI and stirring for 1 h to form the reactive N-acylimidazole intermediate. Subsequently, a pullulan solution (1 g in 20 mL DMSO) was added, and the reaction was continued for 24 h at 70 °C. Nitrogen purging was applied during the first hour of the reaction to minimize the presence of oxygen and moisture. The reaction mixture was precipitated in ethanol and stored at 4 °C overnight. The resulting pullulan esters were washed three times with ethanol and twice with water prior to freeze drying, to remove residual reagents and byproducts. At least two independent synthesis batches were prepared for each pullulan ester formulation to verify reproducibility of the esterification procedure.

### 4.3. Spin Coating of Pullulan Esters

Pullulan esters were dissolved in a chloroform/HFIP (80/20, *v*/*v*) mixture at a concentration of 20 mg/mL and spin-coated onto circular glass coverslips (ϕ 19 mm) using an Ossila spin coater (Ossila, Sheffield, UK). Prior to coating, the substrates were sequentially cleaned with acetone, isopropanol, and Milli-Q water for 10 min each. Immediately before spinning, 100 µL of the solution was deposited onto each coverslip and spun at 6000 rpm for 60 s. The substrates were then placed on a heating plate at 60 °C overnight to remove residual solvent. Optimization of spin-coating parameters was not performed, as these conditions yielded uniform and smooth coatings for all samples.

### 4.4. Characterization Techniques

The obtained pullulan ester samples were systematically characterized to evaluate their chemical, molecular, thermal, structural, and surface properties, as described in the following sections.

#### 4.4.1. Chemical Characterization

The chemical modification of pullulan was confirmed using Fourier Transform Infrared (FTIR) and Proton Nuclear Magnetic Resonance (^1^H-NMR) spectroscopies, as well as elemental analysis. FTIR spectra were recorded using an Alpha II Compact Spectrometer over a range of 4000–400 cm^−1^ with 32 scans. Baseline correction and normalization were performed using Fityk (Version 1.3.1), with normalization to the C-O stretching peak at 1012 cm^−1^.

A Bruker Avance III HD 400 spectrometer was used to acquire ^1^H-NMR spectra at 25 °C. As the pullulan esters were insoluble in chloroform-d, DMSO-d_6_, and deuterium oxide (D_2_O), a mixed solvent system was required. Several chloroform-d/DMSO-d_6_ solvent ratios were evaluated experimentally. A ratio of 60/40 (*v*/*v*) was selected because it provided complete dissolution of all synthesized pullulan esters while maintaining adequate spectral resolution. Therefore, the samples were dissolved in a chloroform-d/DMSO-d_6_ (60/40) solvent mixture at a concentration of 20 mg/mL, while pristine pullulan was dissolved in DMSO-d_6_. Spectra were recorded using a 30° pulse with 16 scans.

Elemental (CH) analysis was performed using a Flash 2000 Organic Elemental Analyzer (Thermo Fisher Scientific, Waltham, MA, USA). The DS was calculated from the measured carbon content using the following equation:

(1) $$C\%=\;\frac{12\;\times \;(6+n\;\times \;DS)}{12\;\times \;\left(6+n\;\times \;DS\right)+\;1\;\times \;\left(10+\left(2n-2\right)\;\times \;DS\right)+16\;\times \;(5+\;DS)}$$

where *C*% represents the experimental carbon weight percentage in the sample, n the number of fatty acid carbons (14 for MA, 16 for PA, 18 for SA), and 1, 12, and 16 the atomic masses of hydrogen, carbon, and oxygen, respectively.

#### 4.4.2. Molecular and Solution Properties

Molecular weight and solution behavior were evaluated to assess the influence of modification on polymer chain properties. The molecular weight was determined by Gel Permeation Chromatography (GPC)/Size Exclusion Chromatography (SEC) in tetrahydrofuran (THF) using an Agilent 1260 Infinity II GPC system equipped with a PL gel MIXED-C column, a refractive index detector (RID), and a UV-Vis detector. The system was calibrated using polystyrene standards. Measurements were performed at a flow rate of 1 mL/min and a temperature of 40 °C. Samples were fully dissolved in THF prior to analysis, filtered through a 0.2 µm PTFE filter, and injected with a volume of 20 µL. Data processing was carried out using Agilent GPC/SEC software (Open Lab).

To assess solution behavior, the viscosity of 20 mg/mL solutions in chloroform/HFIP (80/20, *v*/*v*) was measured using an Anton Paar MCR501 rheometer (Anton Paar Group, Graz, Austria) at 20 °C with a cone-plate geometry (CP50-1/Ti). To minimize solvent evaporation during testing, both a solvent trap and a solvent plate were employed. The sample solution was loaded onto the solvent plate, after which the geometry was immediately lowered and the measurement was initiated within 10 s. The viscosity was monitored at a constant shear rate of 10 s^−1^ for 150 s. Steady-state viscosity values were determined after an initial equilibration period, during which a steady flow profile is established.

#### 4.4.3. Thermal Properties

Thermal stability and transitions were investigated using thermogravimetric analysis (TGA) and differential scanning calorimetry (DSC). TGA measurements were performed on a TGA Q500 (TA Instruments, New Castle, DE, USA) over a temperature range from 20 °C to 550 °C, with a controlled heating rate of 10 °C per minute under nitrogen atmosphere.

DSC measurements were conducted using a DSC Q2000 (TA Instruments). Samples were subjected to repeated heating and cooling cycles between −20 °C and 110 °C at a rate of 10 °C per minute.

#### 4.4.4. Structural Properties

Structural organization was evaluated using X-ray diffraction (XRD) with a PANalytical X’Pert PRO diffractometer (PANalytical B.V., Almelo, The Netherlands), equipped with a Cu Kα radiation source (λ = 1.5406 Å) operated at 45 kV and 40 mA. Measurements were conducted at ambient temperature using continuous scanning with a counting time of 400 s. X-ray data were collected over a 2θ range of 4° to 40° using continuous scanning and subsequently processed with X’Pert Data Viewer (version 1.7) from PANalytical and baseline-corrected with Fityk.

#### 4.4.5. Surface Properties

Surface properties of the pullulan ester coatings were evaluated in terms of wettability, morphology, and topography. Surface wettability was determined using the sessile drop method with an Attension Theta Flex optical tensiometer (Biolin Scientific, Gothenburg, Sweden). Measurements were performed on at least three coated cover glasses per condition, with three droplets per sample. Contact angles were recorded over 100 s (sample rate of 1.1 frames per second), and average and standard deviation values were computed at the 10-s mark.

Surface morphology was examined using field-emission scanning electron microscopy (JSM-7200F) at an acceleration voltage of 2 kV and magnifications of ×100, ×10 000, and ×30 000. Prior to imaging, the samples were sputter-coated with a gold/palladium (Au/Pd) layer employing an Auto Fine Coater (JEOL JFC-1300, JEOL Ltd., Tokyo, Japan).

Atomic force microscopy (AFM) was used to assess surface roughness and film thickness. Morphological imaging was performed using an Agilent 5500 AFM equipped with a MAC III controller (Agilent Technologies, Santa Clara, CA, USA), operating in intermittent contact mode in air over 5 × 5 µm^2^ areas, with MSNL-F tips (f = 120 kHz, k = 0.6 N.m^−1^, tip radius of curvature < 12 nm, Bruker AFM Probes, Camarillo, CA, USA). The thickness of the polymer coatings was determined using a Bruker NanoWizard 5 AFM (Bruker Nano, Santa Barbara, CA, USA) by a scratch (step-height) method, with an AIO-D probe (f = 350 kHz, k = 40 N.m^−1^, tip radius of curvature < 10 nm, Budget Sensors, Sofia, Bulgaria). The polymer film surface was locally scratched with the AFM tip using forces between 2 and 3 μN in contact mode to expose the substrate, followed by morphological imaging of the scratch and surrounding polymer film in Quantitative Imaging (QI™) mode (Bruker Nano GmbH, Berlin, Germany) between 50 and 100 nN. Thickness was calculated from height profiles extracted across the scratched region over a lateral distance of 10 µm, using the height difference between the coating surface and the exposed substrate. For both surface characterization and thickness determination, at least three independent areas per sample were analyzed. AFM morphological images leveling and line-correction, together with line profiles and roughness parameters extraction were performed using Gwyddion (Version 2.71), a free and open-source SPM (scanning probe microscopy) data visualization and analysis program [57].

### 4.5. In Vitro Studies

#### 4.5.1. Cytotoxicity

Cytotoxicity was evaluated according to ISO 10993-5 using NIH3T3 fibroblasts cultured in DMEM supplemented with fetal bovine serum (FBS) under standard conditions (37 °C, 5% CO_2_). Cells were harvested using TrypLE Express Enzyme (with EDTA) and seeded into 48 well plates at a density of 1.1 × 10^4^ cells per well in 400 µL medium. Following 24 h to allow attachment, the culture medium was replaced with extract media derived from the tested materials. Coated cover glasses (ϕ 19 mm), sterilized via ultraviolet exposure for 30 min, were briefly treated with ethanol, washed with phosphate-buffered saline (PBS), rinsed with DMEM, and incubated in DMEM for 24 h at 37 °C. This extraction medium was then applied to the fibroblast cultures for 24 h. Fresh DMEM served as the negative control, while a 20% DMSO solution in DMEM was used as the positive control.

Following exposure, cell viability was evaluated utilizing the MTT assay. A working solution was prepared by combining a 5 mg/mL MTT solution (prepared in PBS) with FBS-free DMEM at a 1:9 ratio, followed by a 4-h incubation. Subsequently, the medium was meticulously aspirated, and the formazan crystals were solubilized in 400 µL of DMSO. An aliquot of 100 µL of this solution was transferred to a 96-well plate, after which the absorbance was measured at 570 nm. Cell viability was expressed as a percentage relative to the negative control cells.

#### 4.5.2. Cell Adhesion Test

Endothelial cells (BAEC/BAOEC) were cultured at 37 °C and 5% CO_2_ in DMEM and detached using TrypLE Express Enzyme-EDTA. Coated substrates were fixed in a 12 well plate, sterilized via UV exposure (254 nm) for 30 min, and washed with PBS prior to use. The endothelial cells were seeded onto the sterile coated coverslips at a density of 1.1 × 10^5^ cells per well in 2 mL of growth medium. Following a 24-h incubation, cell attachment and morphology were observed using an IX83 Inverted Microscope (Olympus, Tokyo, Japan). Cell adhesion was evaluated qualitatively based on visible cell-surface interactions. Flattened, spread cells indicated adhesion, whereas rounded or clustered cells indicated poor or absent adhesion.

#### 4.5.3. Agar Diffusion Test

Antimicrobial activity was qualitatively assessed in triplicate using an agar diffusion method. First, films were prepared via solvent casting by pouring a 60 mg/mL solution of pullulan fatty ester in chloroform/HFIP (80/20, *v*/*v*) into silicone molds, followed by solvent evaporation at 60 °C. Concurrently, LB/agar medium was prepared using commercial LB powder supplemented with 5 g/L agar, while overnight cultures of *E. coli* (Gram-negative) and *S. aureus* (Gram-positive) were grown in standard LB broth. After autoclaving, the LB/agar medium was allowed to cool down to approximately 50 °C, and 25 mL of the culture was added to 500 mL of the LB/agar medium. The mixture was gently swirled to ensure a uniform distribution of the bacteria, poured into Petri dishes and left to solidify, after which pullulan ester films were placed onto the surface of the agar. The plates were then incubated overnight and the antimicrobial activity was qualitatively assessed.

## 5. Conclusions

Pullulan fatty acid esters with tunable hydrophobicity were successfully synthesized via N,N-carbonyldiimidazole (CDI)-mediated esterification, enabling systematic variation in alkyl chain length (C14-C18) and degree of substitution (0.24–0.77). The results demonstrate that alkyl chain length is the primary factor governing molecular organization, thermal behavior, and crystallinity, whereas the degree of substitution acts as a secondary tuning parameter that controls the hydrophobic functionality density and influences solution behavior, coating formation, and surface wettability. While shorter chains (C14–C16) yield predominantly amorphous materials, stearate-modified pullulan (C18) exhibits distinct melting transitions (40–42 °C) and the formation of semicrystalline domains driven by side-chain packing. The degree of substitution further modulates material properties by controlling the density of hydrophobic moieties, affecting intermolecular interactions, solubility, and film formation. Despite only modest variations in solution viscosity across all samples, differences in both viscosity and solubility influence solvent evaporation dynamics during spin coating, resulting in nanostructured coating layers with nanoscale thicknesses (~110–150 nm) and sub-nanometer surface roughness (S_a_ < 1 nm). Surface wettability could be systematically adjusted, with water contact angles ranging from 85° to 105°.

From a functional perspective, all coatings exhibited excellent cytocompatibility, absence of endothelial cell adhesion, and a localized contact-dependent antibacterial effect without evidence of leaching. These findings highlight the potential of pullulan fatty esters as bio-inert, non-fouling surface coatings. Overall, this study establishes clear structure–property relationships linking chemical modification, molecular organization, and thin-film behavior. The ability to systematically adjust the coating structure and surface properties through variation of alkyl chain length and degree of substitution provides a versatile platform for the development of pullulan fatty ester nanocoatings in biomedical and advanced material applications.

## Figures and Tables

**Table 1:** Degree of substitution (DS), weight-average molecular weight (MW_w_), polydispersity index (PDI), and viscosity (η) of pullulan ester solutions (20 mg/mL in chloroform/HFIP, 80/20 *v*/*v*). Pullulan myristate (PME), pullulan palmitate (PPE), and pullulan stearate (PSE) were synthesized at esterification molar ratios (AGU-CDI-fatty acid) of (1-2-2) and (1-3-3).

Sample	Molar Ratio	DS [-]	MW_w_ [Da]	PDI [-]	η [mPa·s]
PME	1-2-2	0.24 ± 0.01 *	5.2 × 10^5^	2.56	2.92 ± 0.21 *
	1-3-3	0.47 ± 0.01 *	6.1 × 10^5^	2.89	3.67 ± 0.11 *
PPE	1-2-2	0.28 ± 0.01 *	7.5 × 10^4^	1.67	1.94 ± 0.15 *
	1-3-3	0.63 ± 0.01 *	2.7 × 10^5^	2.04	2.16 ± 0.04 *
PSE	1-2-2	0.35 ± 0.00 *,**	5.8 × 10^5^	2.86	2.91 ± 0.04 *
	1-3-3	0.77 ± 0.04 *	6.4 × 10^5^	2.90	2.97 ± 0.06 *

* Three samples tested. ** The standard deviation for the DS of PSE (1-2-2) was 0.001, which is below the reporting threshold.

**Table 2:** Thermal behavior of pullulan myristate (PME), pullulan palmitate (PPE), and pullulan stearate (PSE), synthesized with esterification molar ratios (AGU-CDI-fatty acid) of (1-2-2) and (1-3-3). Thermal stability parameters, including degradation onset ($T_{d,onset}$) and inflection ($T_{d,inf}$) temperatures, were determined via thermogravimetric analysis (TGA). Phase transitions, including melting ($T_{m}$) and crystallization ($T_{c}$) temperatures, were identified using differential scanning calorimetry (DSC). Stearate-modified samples exhibit melting transitions indicative of side-chain crystallization.

Sample	Molar Ratio	$T_{d,onset,1}$ [°C]	$T_{d,onset,2}$ [°C]	$T_{d,inf,1}$ [°C]	$T_{d,inf,2}$ [°C]	$T_{m}$ [°C]	$T_{c}$ [°C]
PME	1-2-2	132	314	142	342	-	-
	1-3-3	120	317	134	347	-	-
PPE	1-2-2	94	316	128	348	-	-
	1-3-3	94	329	131	357	-	-
PSE	1-2-2	-	312	-	343	40	21
	1-3-3	-	327	-	359	42	14

**Table 3:** Film thickness (t), mean surface roughness ($S_{a}$), RMS roughness ($S_{q}$), and static water contact angle ($\theta_{w}$) of pullulan myristate (PME), pullulan palmitate (PPE), and pullulan stearate (PSE) coatings at esterification molar ratios (AGU-CDI-fatty acid) of (1-2-2) and (1-3-3). Coating hydrophobicity ($\theta_{w}$) increases with alkyl chain length and degree of substitution. Values are reported as mean ± standard deviation (n = 3 independent measurements).

Sample	Molar Ratio	t [nm]	S_a_ [nm]	S_q_ [nm]	θ_w_ [°]
PME	1-2-2	149 ± 14	0.34 ± 0.02	0.43 ± 0.02	85 ± 7
	1-3-3	121 ± 7	0.34 ± 0.01	0.43 ± 0.01	87 ± 5
PPE	1-2-2	132 ± 56	0.69 ± 0.08	1.01 ± 0.21	87 ± 6
	1-3-3	110 ± 9	0.39 ± 0.03	0.54 ± 0.09	90 ± 5
PSE	1-2-2	129 ± 19	0.37 ± 0.02	0.47 ± 0.02	95 ± 4
	1-3-3	112 ± 4	0.47 ± 0.03	0.59 ± 0.05	105 ± 2

## Data Availability

The original contributions presented in this study are included in the article/Supplementary Materials. Further inquiries can be directed to the corresponding authors.

## Supplementary Materials

The following supporting information can be downloaded at: https://www.mdpi.com/article/10.3390/ijms27177768/s1. References [4,9,11,12,13,15,19,22,24,47,50,54,58,59,60] are cited in the Supplementary Materials.
