# Supplementary material for: Pullulan Fatty Ester Nanocoatings with Tunable Surface Wettability and Bio-Inert Surface Properties
Source: Int J Mol Sci. 2026 Aug 30;27(17):7768. doi: 10.3390/ijms27177768 (PMC13566633; doi:10.3390/ijms27177768)
Supplement: Supplementary file 1 [file ijms-27-07768-s001.zip › ijms-4525295-supplementary.pdf]

Pullulan Fatty Ester Nanocoatings with Tunable Surface Wettability and Bio-Inert Surface Properties

SUPPLEMENTARY INFORMATION

De Ceulaer Femke[a], Chiu Hao-Chun[a], Deschaume Olivier[b], Bartic Carmen[b], Fardim Pedro[a]

[a] *Chemical and Biochemical Reactor Engineering and Safety (CREaS), Department of Chemical Engineering, KU Leuven, Celestijnenlaan 200F, Leuven, 3001, Belgium*

[b] *Soft Matter and Biophysics, Department of Physics and Astronomy, KU Leuven, Celestijnenlaan 200D, Leuven, 3001, Belgium*

1. Gel permeation chromatography (GPC)

1.1 Commercial pullulan

Commercial pullulan was obtained from TCI Europe NV. A multi-detector GPC instrument (Malvern Panalytical, Omnisec software), equipped with a Column A 6000 M (< 20,000,000 g/mol), a refractive index (RI) detector, differential and intrinsic viscosity (DP, IP) detectors, as well as a multi-angle light scattering (MALS) detector, was employed to ascertain the molecular weight distribution (MWD) and the Mark-Houwink parameters. The sample, prepared at a concentration of 2 mg/mL in 0.1 M $NaNO_3$, was filtered through a 0.2 µm cellulose acetate filter prior to injection. The flow rate was regulated at 0.8 mL/min, with the temperature maintained at 35 °C, and the injection volume was set to 100 µL. Measurements were conducted in triplicate.

The number-average molecular weight, weight-average molecular weight, and polydispersity index are displayed in Table S1. A unimodal distribution with a number-average molecular weight (MW_n) of 1.2×10^5 Da and a weight-average molecular weight (MW_w) of 1.6×10^5 Da. This corresponds to a polydispersity index of 1.38, indicating that the commercial pullulan used for synthesizing pullulan fatty esters is moderately polydisperse.

The Mark-Houwink equation is given by

$$[\eta] = K.MW^a$$

where $[\eta]$ is the intrinsic viscosity, MW is the molecular weight, and K and a are the Mark-Houwink constants. The Mark-Houwink parameters a and $\log K$ were determined to be 0.62 and -3.47 dL/g (corresponding to a K = 3.39 x 10^{-4} mL/g). An a-value of 0.62 indicates that the polymer is structured in random coils, while a $\log K$ value of -3.47 dL/g is consistent with values reported for biopolymeric aqueous solutions. Because K is highly dependent on the polymer type, temperature, and solvent, previously reported K values for pullulan range from 16.6 x 10^3 mL/g in 0.1 M $NaNO_3$ at 20 °C to 1.79 x 10^{-4} - 24.66 x 10^{-4} mL/g in various aqueous solutions at different temperatures [1–3].

1.2 Pullulan fatty esters

GPC analysis was performed on the synthesized pullulan fatty esters using an Agilent 1260 Infinity II GPC system, equipped with a PL gel MIXED-C column, refractive index (RI) detector and UV-VIS detector. The sample, prepared at a concentration of 2 mg/mL in THF, was filtered through a 0.2 µm PTFE filter prior to injection. The flow rate was regulated at 1 mL/min, with the temperature maintained at 40 °C, and the injection volume was set to 20 µL. The molecular weight parameters are provided in Table S1.

Table S1. Multi-detector GPC results for commercial pullulan and pullulan esters (PME, PPE, PSE), including number-average molecular weight (MW_n), weight-average molecular weight (MW_w), and polydispersity index (PDI).

	Comm. Pullulan**	PME 122*	PME 133*	PPE 122*	PPE 133*	PSE 122*	PSE 133*
**MW_n (Da)**	$1.2x10^5$	$2.0x10^5$	$2.1x10^5$	$4.5x10^4$	$1.3x10^5$	$2.0x10^5$	$2.2x10^5$
**MW_w (Da)**	$1.6x10^5$	$5.2x10^5$	$6.1x10^5$	$7.5x10^4$	$2.7x10^5$	$5.8x10^5$	$6.4x10^5$
**PDI**	1.38	2.56	2.89	1.67	2.04	2.86	2.90

* Two samples tested; ** Three samples tested.

2. Reaction scheme CDI-mediated esterification of pullulan with fatty acids

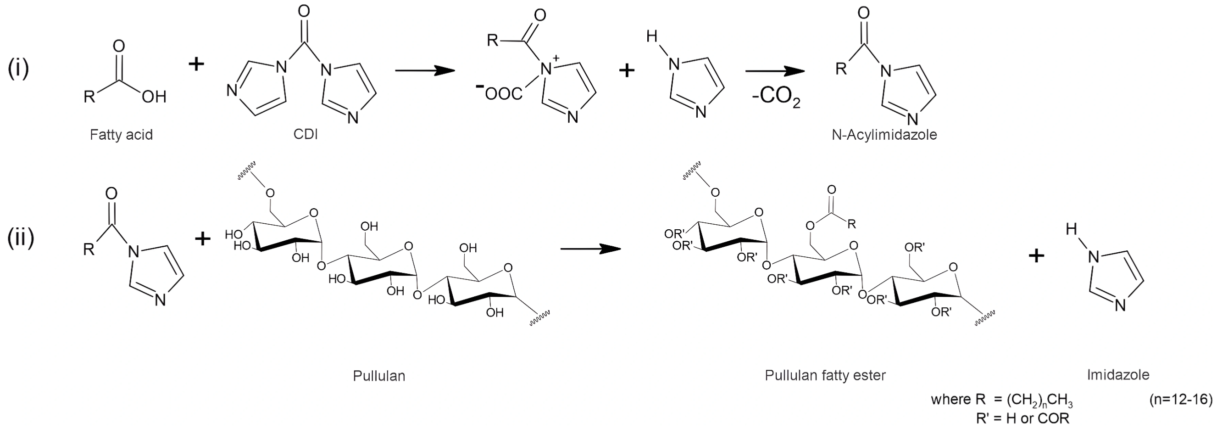

Figure S1. Schematic overview of the two-step synthesis of pullulan-fatty acid esters via N-acylimidazole intermediates. (i) The activation of the fatty acid (with alkyl chain length n) using N,N-carbonyldiimidazole (CDI) to generate the reactive N-acylimidazole intermediate, with the concomitant release of carbon dioxide and imidazole. (ii) The esterification of the hydroxyl groups of pullulan with the N-acylimidazole intermediate, resulting in the formation of pullulan-fatty acid esters and the by-product imidazole.

3. Fourier Transform Infrared (FTIR) analysis

3.1 FTIR data of pullulan palmitate and pullulan stearate

The FTIR spectra of pullulan palmitate (PPE) and pullulan stearate (PSE) are presented in Figure S2. Similar to the spectrum of pullulan myristate (PME), new peaks appear corresponding to the C=O (ester) stretching at 1726 cm^{-1} and the asymmetric and symmetric C-H stretching around 2920 cm^{-1} and 2849 cm^{-1}, respectively. A decrease in the O-H stretching band around 3400 cm^{-1} is observed for PPE and PSE at different degrees of substitution, reflecting the conversion of hydroxyl groups into ester moieties. Following esterification, the C=O (carbonyl) stretching peak of the fatty acid at 1699 cm^{-1} is replaced by the C=O (ester) stretching peak at 1726 cm^{-1}, confirming successful esterification [4–7].

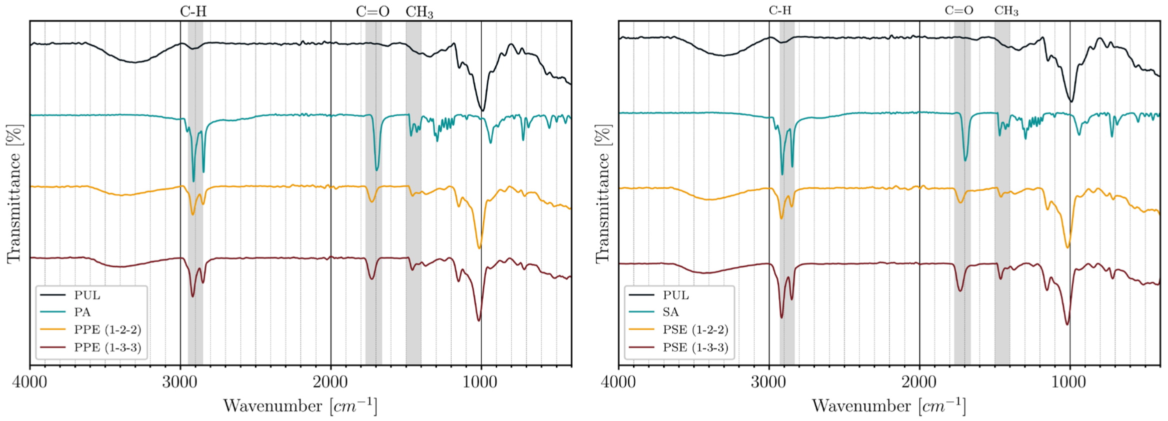

Figure S2. FTIR spectra of pullulan (PUL), palmitic acid (PA), pullulan palmitate (PPE), stearic acid (SA), and pullulan stearate (PSE) at esterification molar ratios (AGU-CDI-fatty acid) of (1-2-2) and (1-3-3). The spectra highlights the C-H stretching, C=O stretching, and characteristic vibrations of the methyl (CH_3) groups. A clear shift of the C=O (carbonyl) stretching peak from PA and SA to the corresponding ester C=O peak in PPE and PSE confirms successful esterification.

3.2 FTIR data of pullulan ester films after chloroform/HFIP evaporation at 60 °C

To study structural stability during spin coating, a similar procedure was used to produce pullulan ester films, thereby facilitating subsequent ATR-FTIR characterization. Briefly, 60 mg/mL pullulan ester solutions in chloroform/HFIP (80/20, v/v) were prepared and poured into 1.5 cm x 1.5 cm square molds. These molds were placed on a heating plate at 60 °C to evaporate the chloroform/HFIP solvent overnight. Transparent thin films were obtained and subjected to ATR-FTIR analysis (Figure S3).

As shown in Figure S4, the C=O (ester) peaks at 1726 cm^{-1} and the asymmetric and symmetric C-H stretching peaks around 2920 cm^{-1} and 2849 cm^{-1} remain intact after heating and solvent removal for all pullulan-fatty acid formulations [4,6,7]. Therefore, the pullulan esters retain their functional groups after processing because the heating plate temperature (60 °C) during chloroform/HFIP evaporation is well below the onset of thermal degradation, as determined by TGA analysis (Section 3.4). The lowest onset of degradation for the first degradation step, corresponding to the cleavage of fatty acid ester groups, is observed for PPE (1-2-2) and (1-3-3), whereas degradation of the palmitic acid side chains starts at 94 °C.

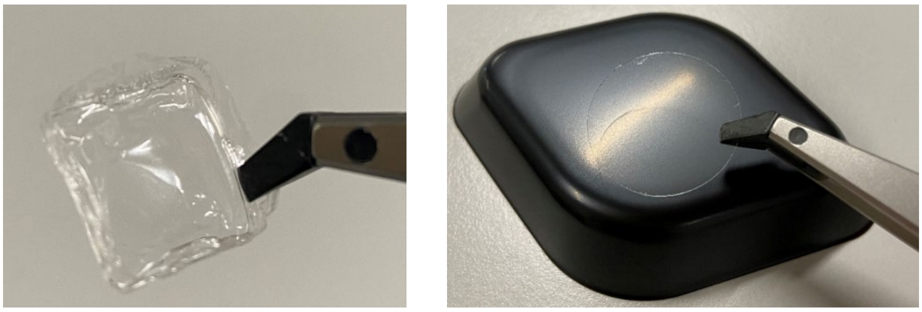

Figure S3. Representative images of pullulan myristate (PME, 1-2-2) after solvent evaporation from a chloroform/HFIP (80/20, v/v) solution. The left image shows a self-supported solvent-cast film obtained after evaporation of the solvent mixture. The right image shows a spin-coated PME (1-2-2) coating deposited on a glass coverslip, representative of the substrates used for SEM, AFM, contact angle, and biological experiments.

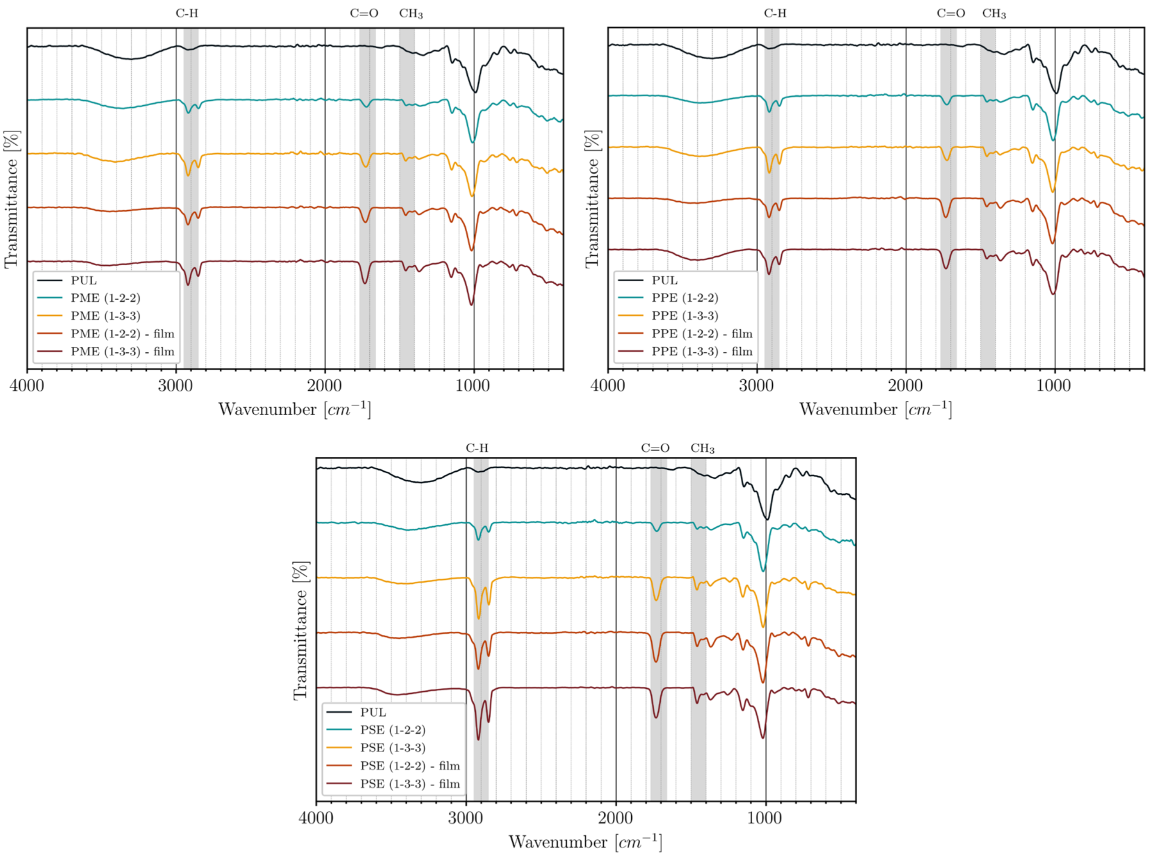

Figure S4. FTIR spectra of pullulan (PUL), pullulan myristate (PME), pullulan palmitate (PPE), and pullulan stearate (PSE) before and after film formation. The pullulan fatty esters are synthesized with the esterification molar ratios (AGU-CDI-fatty acid) of (1-2-2) and (1-3-3). The spectra highlights the C-H stretching, C=O stretching, and characteristic vibrations of the methyl (CH_3) groups.

4. NMR analysis of pullulan-fatty esters (pullulan palmitate and pullulan stearate)

4.1 Hydrogen-bonded complex of chloroform-DMSO systems

The hydrophobic pullulan esters have a hydrophilic backbone and a hydrophobic side chain. Therefore, a solvent mixture of chloroform-d/DMSO-d_6 (60/40, v/v) was selected to dissolve the hydrophobic pullulan-fatty acid esters, since they are insoluble in chloroform, DMSO and water individually. The chloroform-d/DMSO-d_6 (60/40, v/v) mixture provides a medium-polarity, amphiphile-compatible solvent environment and forms a hydrogen-bonded complex between chloroform-d and DMSO-d_6 that is less polar than DMSO-d_6 and more polar than chloroform-d.

Chloroform-d contains three chlorine ions that reduce the electron density of the central carbon atom, increasing the acidity of the C-H proton enough to be activated by weak hydrogen bond acceptors such as the oxygen in the S=O bond in DMSO-d_6. This bond is strongly polarized, and the oxygen atom carries a substantial partial negative charge, making it an extremely powerful Lewis base. When chloroform-d and DMSO-d_6 are mixed, a dipole-dipole interaction takes place where the electron cloud around the proton of chloroform is shifted toward the sulfoxide oxygen. This reduces shielding of the proton, causing a downfield shift of the chloroform-d peak in the ^{1}H-NMR spectrum of PME, PPE and PSE. In this complex system, the hydrogen-bonding interactions are only slightly greater than typical van der Waals interactions [8,9].

The dissociation of the chloroform-d/DMSO-d_6 complex is given by

$$Cl_3CD \ldots O = S(CD_3)_2 \underset{k_f}{\overset{k_d}{\rightleftharpoons}} CDCl_3 + O = S(CD_3)_2$$

where k_d and k_f are the dissociation and formation rate constants of the hydrogen-bonded complex formed between chloroform-d and DMSO-d_6 [8].

4.2 NMR spectra of pullulan palmitate and pullulan stearate

The ^{1}H-NMR spectra of pullulan, pullulan palmitate, and pullulan stearate depicted in Figure S5 exhibit trends analogous to those observed for pullulan myristate in Figure 2. All the modified pullulan esters display a peak corresponding to the terminal methyl group at 0.87 ppm, associated with H22 of pullulan palmitate and H24 of pullulan stearate. The protons of the alkyl groups, identified as H9-H21 for pullulan palmitate and H9-H23 for pullulan stearate, resonate at 1.25 ppm and 1.57 ppm, respectively, while the H8 proton adjacent to the ester linkage appears at 2.38 ppm. The H2-H6 protons of the anhydroglucose unit (AGU) resonate between 3.10 ppm and 4.20 ppm. In addition, the anomeric protons (H1), situated near the α-(1,4)- and α-(1,6) glycosidic bonds, along with hydroxyl protons, are detected within the range of 4.20 to 5.70 ppm. The solvent proton peaks of DMSO-d_6 and chloroform-d are observed at 2.56 ppm and 7.98 ppm, respectively. Residual water present in the sample or DMSO-d_6 is noted at 3.36 ppm [4,6,10,11].

Normalization to the terminal methyl peak (H22 of pullulan palmitate and H24 of pullulan stearate) allows for direct comparison of the pullulan-fatty acid ester formulations. For PPE (1-3-3) and PSE (1-3-3), the intensities of the H8 protons, protons of the pullulan backbone, and anomeric protons are lower than those of PPE (1-2-2) and PSE (1-2-2), respectively. The fatty acid side chain signals are therefore more dominant at higher molar ratios, indicating that increasing the CDI and fatty acid concentrations results in a higher degree of substitution.

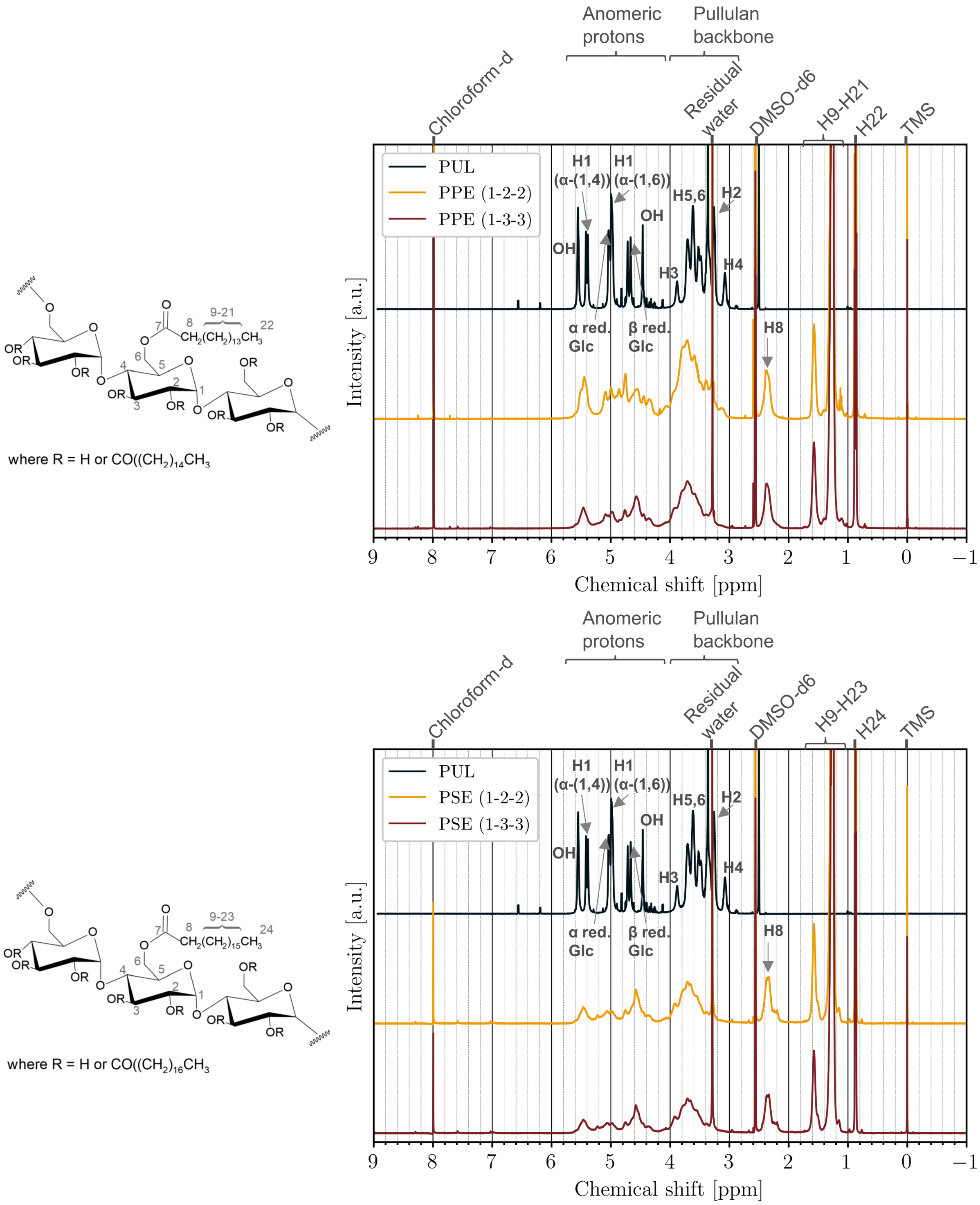

Figure S5. Chemical structure of pullulan palmitate (PPE) and pullulan stearate (PSE) and corresponding ^{1}H-NMR of pullulan in DMSO-d_6 compared with PPE and PSE in chloroform-d/DMSO-d_6 (60/40, v/v), synthesized at esterification molar ratios (AGU-CDI-fatty acid) of (1-2-2) and (1-3-3). The spectra highlights the protons of the pullulan backbone and the emergence of characteristic aliphatic proton resonances (H8-H22) and (H8-H24) associated with palmitoyl and stearoyl moieties, confirming successful esterification. For the PPE and PSE samples, spectra were normalized to the terminal methyl signal of the fatty acid (~0.87 ppm), allowing comparison of substitution levels between PPE and PSE samples.

5. Viscosity profiles of pullulan ester solutions

The viscosity profiles of pullulan ester solutions in chloroform/HFIP (80/20, v/v) and a blank chloroform/HFIP mixture are illustrated in Figure S6. An initial decrease in the viscosity is observed for all samples, however, this effect is particularly observed for PME 122 and PPE 122. This is likely the result of structural relaxation of the polymer chains and alignment under shear. Minor redistribution of the sample within the measuring geometry at the onset of rotation may contribute to the initial decrease, but this effect is dominated by shear-induced equilibration. After approximately 30 seconds, a steady flow profile is established for all samples, and the steady-state viscosity values are determined over three time windows (30-50 s, 50-100 s, 100-150 s), as displayed in Table S2. The blank chloroform/HFIP mixture showed a viscosity of 0.87 mPa·s between 50-100 s.

Therefore, it can be stated that evaporation of chloroform/HFIP is limited for the tested timeframe (< 150 s) using a solvent trap and fast handling after sample loading.

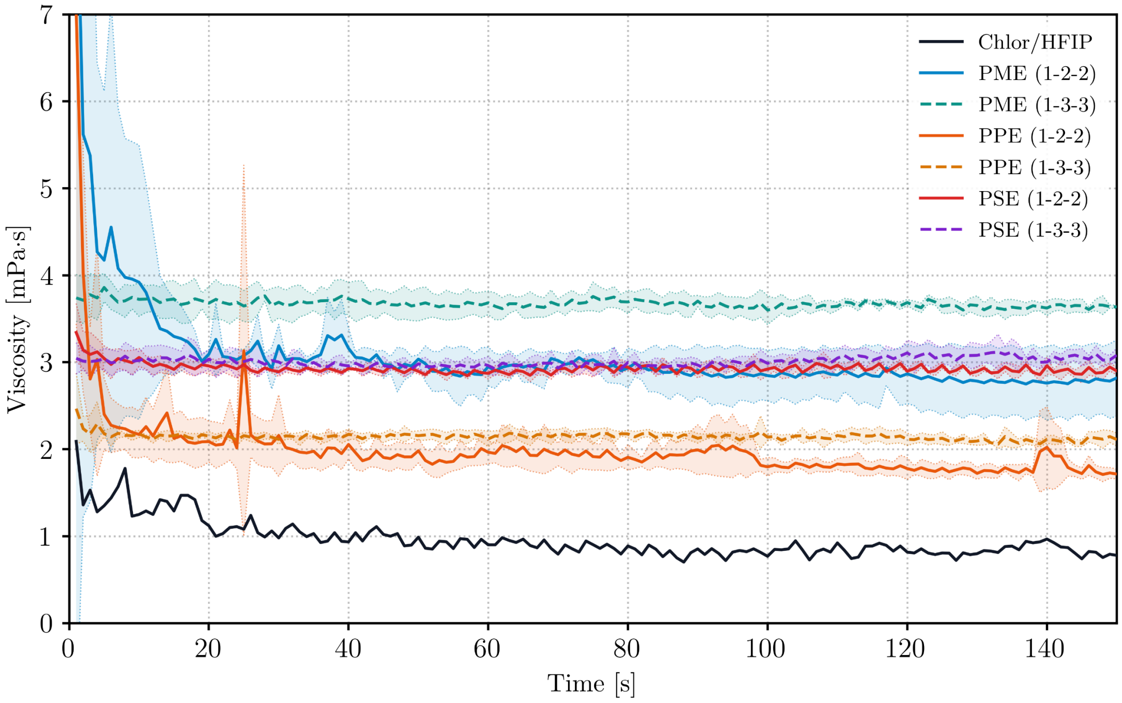

Figure S6. Viscosity profiles of 20 mg/mL pullulan ester solutions in chloroform/HFIP (80/20, v/v), alongside the pure chloroform/HFIP (80/20, v/v) solvent mixture (blank) for comparison.

Table S2. Average viscosity values of 20 mg/mL solutions of pullulan myristate (PME), pullulan palmitate (PPE), and pullulan stearate (PSE) in chloroform/HFIP (80/20, v/v), evaluated across three time windows (30-50 s, 50-100 s, and 100-150 s). Values represent mean ± standard deviation (n = 3).

SAMPLE	MOLAR RATIO	VISCOSITY [mPa·s] 30-50 s	VISCOSITY [mPa·s] 50-100 s	VISCOSITY [mPa·s] 100-150 s
PME	1-2-2	3.07 ± 0.06	2.92 ± 0.21	2.82 ± 0.36
	1-3-3	3.69 ± 0.16	3.67 ± 0.11	3.65 ± 0.05
PPE	1-2-2	1.96 ± 0.16	1.94 ± 0.15	1.79 ± 0.05
	1-3-3	2.15 ± 0.03	2.16 ± 0.04	2.12 ± 0.03
PSE	1-2-2	2.91 ± 0.03	2.91 ± 0.04	2.92 ± 0.04
	1-3-3	2.97 ± 0.07	2.97 ± 0.06	3.05 ± 0.08

6. Thermal characterization of pure fatty acids and pullulan fatty esters

6.1 Fatty acids

A Thermogravimetric (TGA Q500, TA Instruments) and Differential Scanning Calorimetry (DSC Q2000, TA Instruments) analysis was performed for myristic acid (MA), palmitic acid (PA), and stearic acid (SA), as shown in Figure S7. The onset of degradation (defined by a 5% weight loss) and the corresponding inflection points were determined to be 166 °C and 225 °C for myristic acid (C14), 183 °C and 242 °C for palmitic acid (C16), and 200 °C and 261 °C for stearic acid (C18). In the DSC thermograms, the heating cycles of myristic acid, palmitic acid, and stearic acid are presented, showing melting points of 56 °C, 64 °C, and 70 °C, respectively. An increase in the alkyl chain length, represented by n, leads to a higher onset of degradation, inflection point and melting point, indicating that longer fatty acid chains exhibit higher thermal stability [12,13].

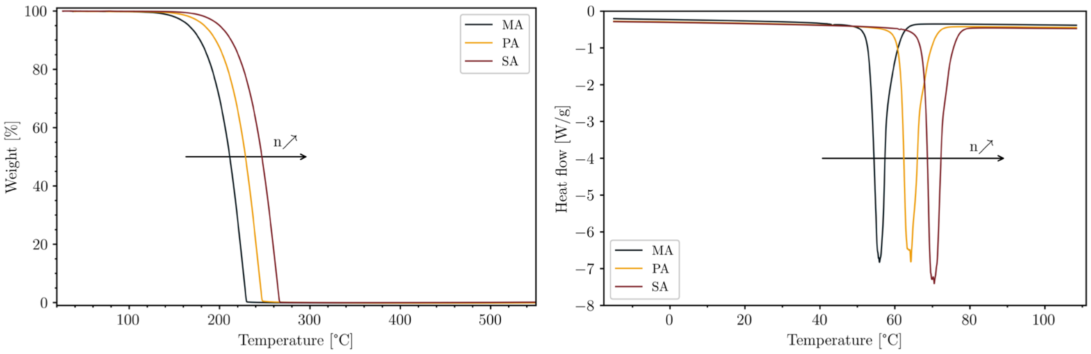

(a) (b)

Figure S7. Thermal characterization of fatty acids with varying alkyl chain length (C14-C18): (a) TGA analysis of myristic acid (MA), palmitic acid (PA), and stearic acid (SA). (b) DSC analysis of myristic acid (MA), palmitic acid (PA), and stearic acid (SA). Increasing the alkyl chain length results in higher degradation temperatures and higher melting points. For the DSC analysis, only the heating cycle is shown. Overall, fatty acids with longer chain lengths (n) exhibit increased thermal stability.

6.2 Pullulan fatty esters

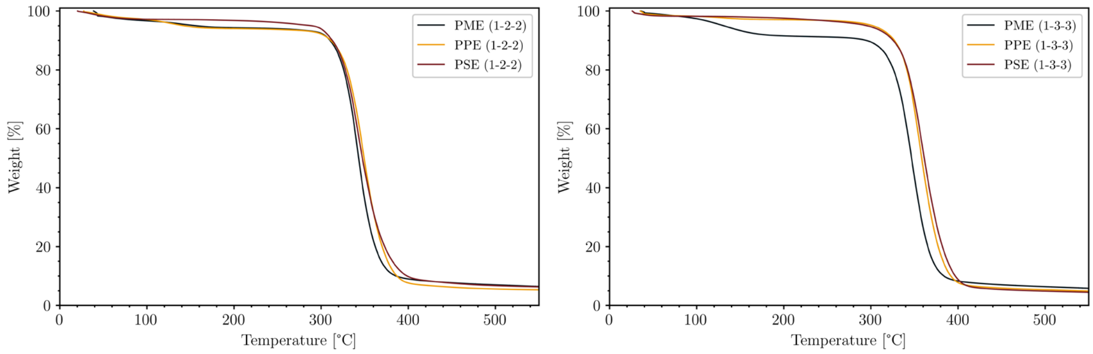

(a) (b)

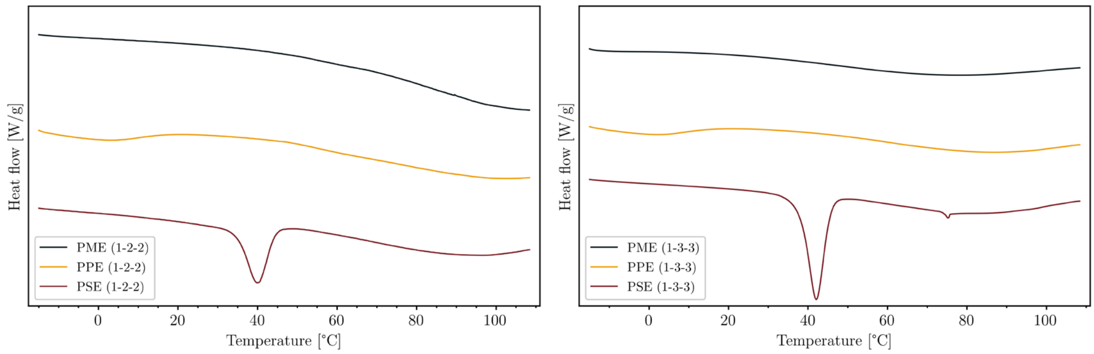

(c) (d)

Figure S8. Thermal characterization of pullulan myristate (PME), pullulan palmitate (PPE), and pullulan stearate (PSE): (a) TGA analysis for the (1-2-2) formulations; (b) TGA analysis for the (1-3-3) formulations; (c) DSC analysis for the (1-2-2) formulations; (d) DSC analysis for the (1-3-3) formulations. Increasing the alkyl chain length results in a higher thermal stability, and induces melting and crystallization behavior.

7. SEM analysis of spin-coated pullulan ester films

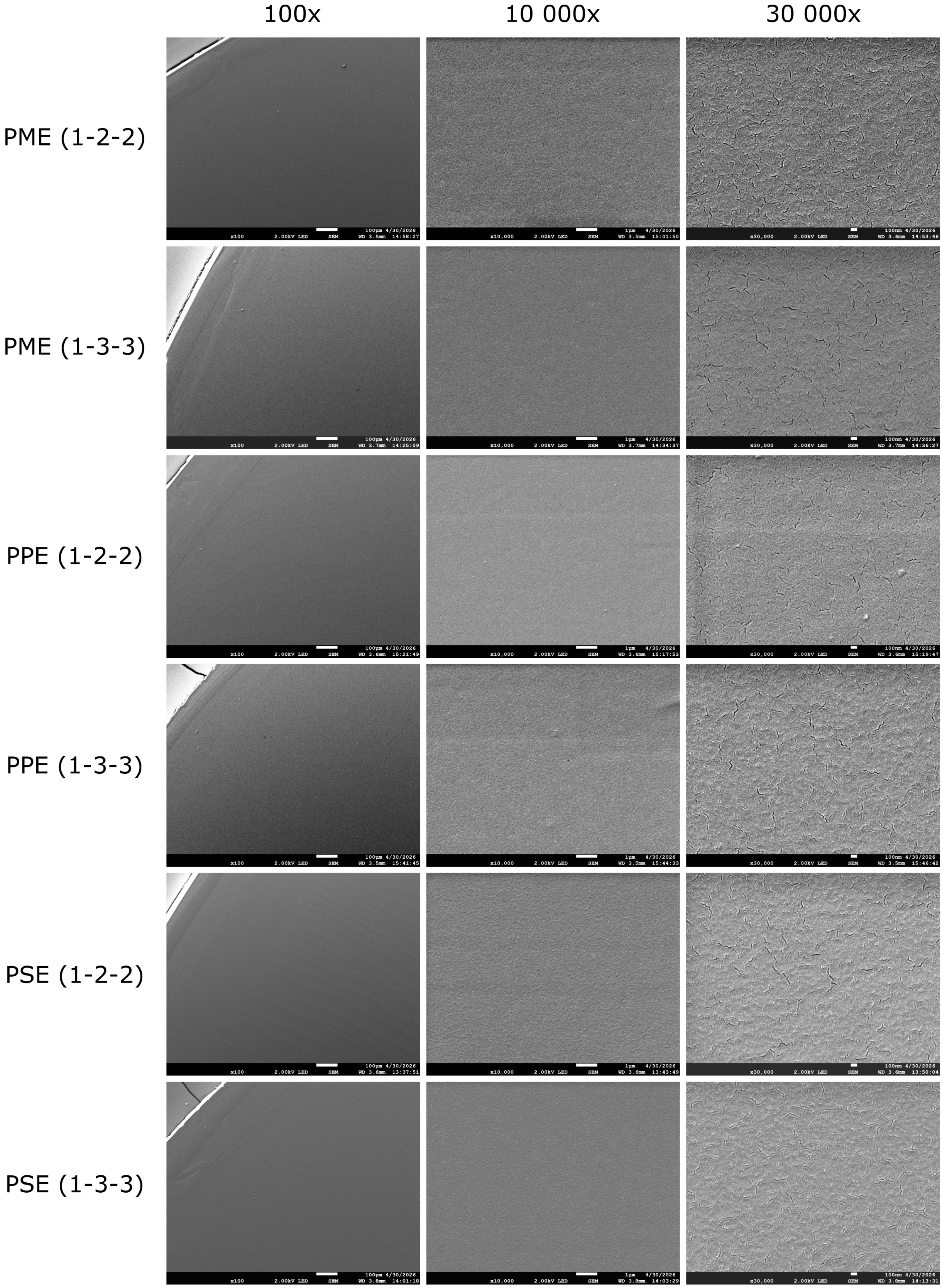

Figure S9. SEM images of the surface of pullulan myristate (PME), pullulan palmitate (PPE), and pullulan stearate (PSE) films. All coatings exhibit smooth and homogeneous surfaces at low (x100) and intermediate (x10,000) magnifications, while fine crack networks are observed at the highest magnification (x30,000). Scale bars correspond to 100 µm, 1 µm, and 100 nm, respectively.

8. AFM analysis of spin-coated pullulan ester films

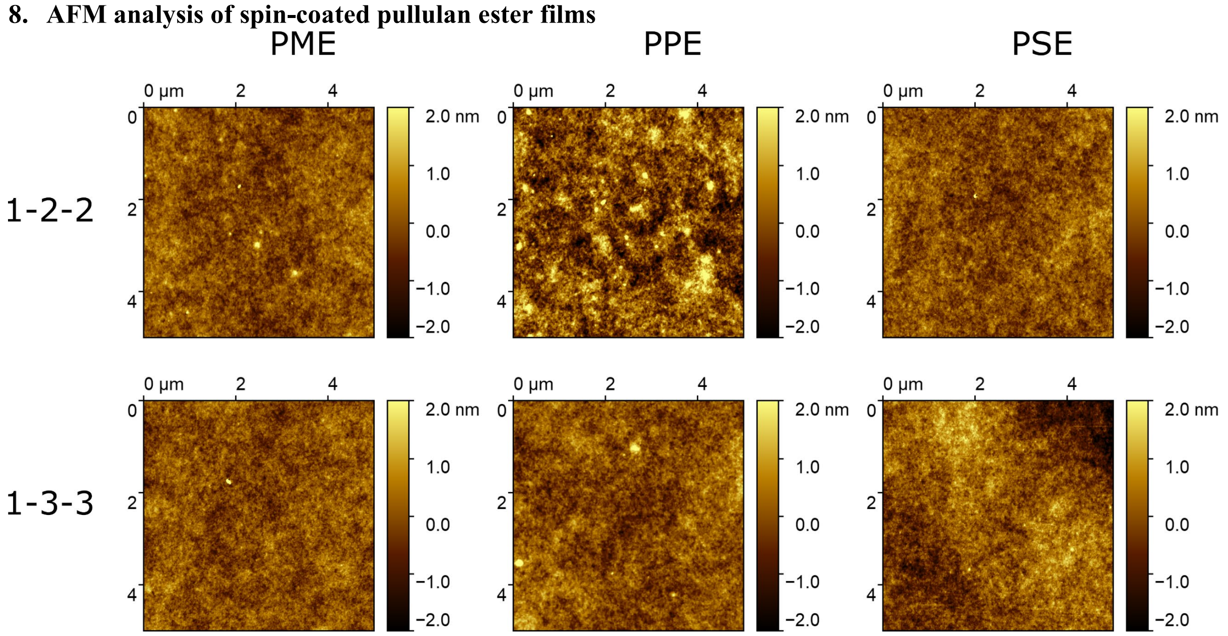

Figure S10. AFM height images of spin-coated pullulan myristate (PME), pullulan palmitate (PPE), and pullulan stearate (PSE), synthesized at esterification molar ratios (AGU-CDI-fatty acid) of (1-2-2) and (1-3-3). The images display the nanoscale surface topography and film homogeneity of the respective coatings across 5x5 µm^2 scan areas.

9. Contact angle measurements

An optical tensiometer (Attension Theta Flex, Biolin Scientific) was used to measure the water contact angle at 20 °C in air on a spin-coated pullulan-fatty acid ester coverslip (φ 19 mm). The total measurement duration was set to 100 s at a recording rate of 1.1 frames/s. A direct comparison with a native pullulan film was not performed. Unlike the pullulan esters, native pullulan requires a different solvent system for film preparation and remains highly water soluble. Consequently, swelling and partial dissolution during contact angle measurements may affect the reliability of the measured value and limit direct comparison with the hydrophobically modified pullulan ester coatings.

Based on the images in Figure S11, taken after 10 s, an increase in the water contact angle is observed with increasing alkyl chain length of the fatty acid and with higher molar ratios of CDI to fatty acid during esterification. All samples exhibit contact angles close to 90°. While PME (1-2-2), PME (1-3-3), and PPE (1-2-2) remain slightly hydrophilic, the coatings of PPE (1-3-3), PSE (1-2-2), and PSE (1-3-3) are more hydrophobic. These results demonstrate that, by varying the alkyl chain length and the esterification conditions, pullulan-based coatings with tunable hydrophilicity/hydrophobicity can be prepared [6,14,15].

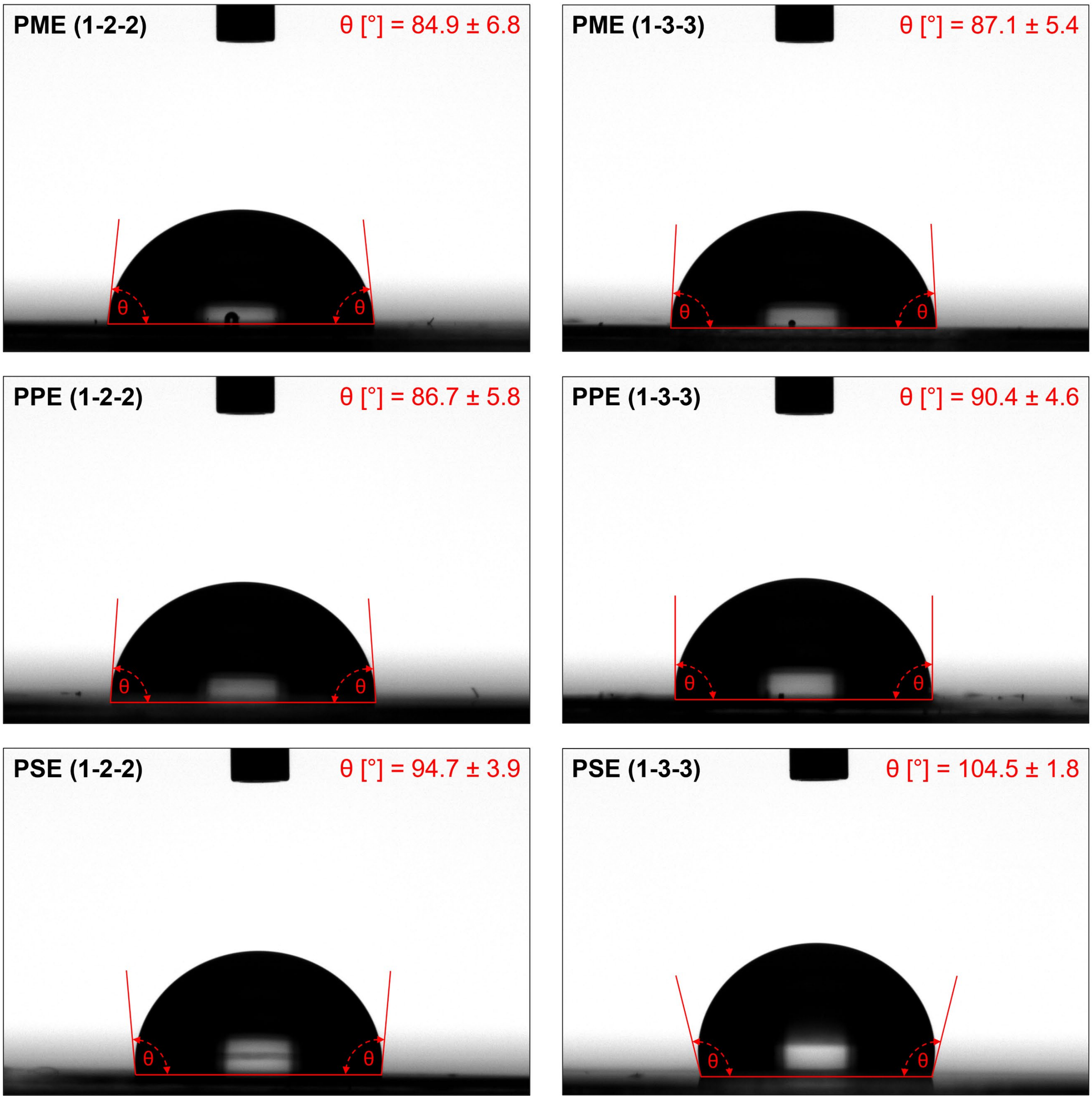

Figure S11. Water contact angle measurements of pullulan fatty ester coatings at 20 °C. Circular glass coverslips (φ 19 mm) were coated with pullulan myristate (PME), pullulan palmitate (PPE), and pullulan stearate (PSE) with esterification molar ratios (AGU-CDI-fatty acid) of (1-2-2) and (1-3-3). The measured static contact angles (θ_w) are shown in red for each sample. For comparison, the contact angle on an uncoated glass coverslip is equal to 27.5 ± 9.1°.

References

1. Butler E. PolymerizeHub Available online: https://www.polychemsci.com/posts/unlocking-polymer-structure-the-markhouwink-equation-in-biomedical-research-and-drug-development (accessed on 2 April 2026).

2. Kasaai, M.R. Intrinsic Viscosity-Molecular Weight Relationship and Hydrodynamic Volume for Pullulan. *J. Appl. Polym. Sci.* **2006**, *100*, 4325–4332, doi:10.1002/APP.22324.

3. Malvern Panalytical What Is the Mark-Houwink Equation? With Examples | Malvern Panalytical Available online: https://www.malvernpanalytical.com/en/learn/knowledge-center/insights/what-is-the-mark-houwink-equation-and-how-do-you-use-it-for-polymer-characterization (accessed on 2 April 2026).

4. De Wever, P.; Van Ostaeyen, B.; Kargl, R.; Kleinschek, K.S.; Fardim, P. Synthesis and Characterization of Dextran Palmitate for Extrusion 3D Printing. *Int. J. Biol. Macromol.* **2025**, *294*, 139399, doi:10.1016/J.IJBIOMAC.2024.139399.

5. Kim, I.S.; Oh, I.J. Preparation and Characterization of Stearic Acid-Pullulan Nanoparticles. *Archives of Pharmacal Research 2010 33:5* **2010**, *33*, 761–767, doi:10.1007/S12272-010-0516-7.

6. Tchobanian, A.; Ceyssens, F.; Cóndor Salgado, M.; Van Oosterwyck, H.; Fardim, P. Patterned Dextran Ester Films as a Tailorable Cell Culture Platform. *Carbohydr. Polym.* **2021**, *252*, 117183, doi:10.1016/J.CARBPOL.2020.117183.

7. Yang, X.; Yuan, Y.; Zhang, N.; Cao, X.; Liu, C. Preparation and Properties of Myristic–Palmitic–Stearic Acid/Expanded Graphite Composites as Phase Change Materials for Energy Storage. *Solar Energy* **2014**, *99*, 259–266, doi:10.1016/J.SOLENER.2013.11.021.

8. Kwak, K.; Rosenfeld, D.E.; Chung, J.K.; Fayer, M.D. Solute-Solvent Complex Switching Dynamics of Chloroform between Acetone and Dimethylsulfoxide - 2D IR Chemical Exchange Spectroscopy. *J. Phys. Chem. B* **2008**, *112*, 13906, doi:10.1021/JP806035W.

9. Daniel, D.C.; McHale, J.L. Hydrogen Bonding in CHCl3/DMSO-D6 and CDCl3/DMSO-H6 Mixtures. *Journal of Physical Chemistry A* **1997**, *101*, 3070–3077, doi:10.1021/JP9623507.

10. Chen, L.; Wang, X.; Ji, F.; Bao, Y.; Wang, J.; Wang, X.; Guo, L.; Li, Y. New Bifunctional-Pullulan-Based Micelles with Good Biocompatibility for Efficient Co-Delivery of Cancer-Suppressing P53 Gene and Doxorubicin to Cancer Cells. *RSC Adv.* **2015**, *5*, 94719–94731, doi:10.1039/C5RA17139C.

11. Van den Eynde, K.; Boon, V.; Gaspar, R.C.; Fardim, P. Biofabrication of Functional Pullulan by Aureobasidium Pullulans under the Effect of Varying Mineral Salts and Sugar Stress Conditions. *Molecules 2023, Vol. 28,* **2023**, *28*, doi:10.3390/MOLECULES28062478.

12. Duchatel-Crépy, L.; Joly, N.; Martin, P.; Marin, A.; Tahon, J.F.; Lefebvre, J.M.; Gaucher, V. Substitution Degree and Fatty Chain Length Influence on Structure and Properties of Fatty Acid Cellulose Esters. *Carbohydr. Polym.* **2020**, *234*, 115912, doi:10.1016/J.CARBPOL.2020.115912.

13. Crépy, L.; Miri, V.; Joly, N.; Martin, P.; Lefebvre, J.M. Effect of Side Chain Length on Structure and Thermomechanical Properties of Fully Substituted Cellulose Fatty Esters. *Carbohydr. Polym.* **2011**, *83*, 1812–1820, doi:10.1016/J.CARBPOL.2010.10.045.

14. Aras, O.; Baydır, E.; Akman, B. Effect of Pre-Anodizing, Electrocoating Processes and Fatty Acid Chain Length on Morphology, Roughness and Durability of ZnO-Coated Superhydrophobic Surfaces. *Applied Physics A 2022 128:3* **2022**, *128*, 176-, doi:10.1007/S00339-022-05303-8.

15. Heale, F.L.; Page, K.; Wixey, J.S.; Taylor, P.; Parkin, I.P.; Carmalt, C.J. Inexpensive and Non-Toxic Water Repellent Coatings Comprising SiO 2 Nanoparticles and Long Chain Fatty Acids. *RSC Adv.* **2018**, *8*, 27064–27072, doi:10.1039/C8RA04707C.
